# Edelfosine-induced metabolic changes in cancer cells that precede the overproduction of reactive oxygen species and apoptosis

**DOI:** 10.1186/1752-0509-4-135

**Published:** 2010-10-06

**Authors:** Vitaly A Selivanov, Pedro Vizán, Faustino Mollinedo, Teresa WM Fan, Paul WN Lee, Marta Cascante

**Affiliations:** 1 Department of Biochemistry and Molecular Biology, Faculty of Biology, Institute of Biomedicine of University of Barcelona (IBUB) and IDIBAPS, Unit Associated with CSIC, 08028 Barcelona, Spain; 2 A.N.Belozersky Institute of Physico-Chemical Biology, Moscow State University, 199899 Moscow, Russia; 3 Centro de Investigación del Cáncer, Instituto de Biología Molecular y Celular del Cáncer, Consejo Superior de Investigaciones Científicas (C.S.I.C.)-Universidad de Salamanca. E-37007 Salamanca, Spain; 4 Department of Chemistry, Center for Regulatory and Environmental Analytical Metabolomics, University of Louisville, Louisville, KY 40292, USA; 5 Department of Medicine, Structural Biology Program, James Graham Brown Cancer Center, University of Louisville, Louisville, KY 40202, USA; 6 Department of Pediatrics, Research and Education Institute, Harbor-UCLA Medical Center, 90502 Torrance, California, USA; 7 Laboratory of Developmental Signalling, Cancer Research UK London Research Institute, 44 Lincoln's Inn Fields, London WC2A 3PX, UK

## Abstract

**Background:**

Metabolic flux profiling based on the analysis of distribution of stable isotope tracer in metabolites is an important method widely used in cancer research to understand the regulation of cell metabolism and elaborate new therapeutic strategies. Recently, we developed software Isodyn, which extends the methodology of kinetic modeling to the analysis of isotopic isomer distribution for the evaluation of cellular metabolic flux profile under relevant conditions. This tool can be applied to reveal the metabolic effect of proapoptotic drug edelfosine in leukemia Jurkat cell line, uncovering the mechanisms of induction of apoptosis in cancer cells.

**Results:**

The study of ^13^C distribution of Jukat cells exposed to low edelfosine concentration, which induces apoptosis in ≤5% of cells, revealed metabolic changes previous to the development of apoptotic program. Specifically, it was found that low dose of edelfosine stimulates the TCA cycle. These metabolic perturbations were coupled with an increase of nucleic acid synthesis *de novo*, which indicates acceleration of biosynthetic and reparative processes. The further increase of the TCA cycle fluxes, when higher doses of drug applied, eventually enhance reactive oxygen species (ROS) production and trigger apoptotic program.

**Conclusion:**

The application of Isodyn to the analysis of mechanism of edelfosine-induced apoptosis revealed primary drug-induced metabolic changes, which are important for the subsequent initiation of apoptotic program. Initiation of such metabolic changes could be exploited in anticancer therapy.

## Background

The characterization of metabolic flux profile in living cells is an important issue in understanding the regulation of normal metabolism and the development of disease processes. Such characterization is then necessary for the development of novel therapeutic strategies. Stable isotope tracing using [1,2-^13^C_2_]-glucose as a source of carbon, has been described as a very powerful tool for metabolic flux profiling [1-6]. The specific pattern of various ^13^C isotopic isomers (isotopomers) fractions measured using mass spectrometry or nuclear magnetic resonance techniques characterized the distribution of metabolic fluxes in the cells under the studied conditions. To evaluate the flux distribution from measured isotopomer distribution a special software tool is necessary.

Classical ^13^C metabolic flux analysis (MFA) evaluated steady state metabolic fluxes based on isotopomer fractions measured under the conditions of isotopic steady state [7]. For non-stationary metabolic flux analysis we developed a tool called "Isodyn" (from "isotopomer dynamics") [8-10] that simulates ^13^C redistribution in metabolites by automatically constructing and solving large systems of differential equations for isotopomers. Although intracellular metabolites could reach isotopic steady state in a range of minutes, the existence of intracellular stores essentially delays the time necessary for establishing isotopic steady state. Such stores as glycogen, aminoacids and lipids, which intensively exchange with intermediates of central carbohydrate metabolism, could prolong the pre-steady state phase for all isotopomers. Of course, there is always a possibility of measuring the labeling of such stores and apply classical ^13^C-MFA for the "fast" intermediates of central metabolism considering that they are in quasi-steady state. However, the simulation of such "slow" variables provides additional information for the determination of the characteristics of the system.

Moreover, there is another reason for using non-stationary analysis based on a kinetic model of considered pathways: it allows, when experimental data is enough, a more profound analysis of kinetic characteristics and regulation in the pathway. Such advantages stimulated the development of other bioinformatic tools for non-stationary flux analysis [11]. Here, an application of Isodyn for revealing the characteristics of cancer cell metabolism and their change induced by a proapoptotic agent edelfosine is described.

Apoptosis is a programmed cell death and the evasion of apoptotic programm is one of the most fundamental characteristics of cancer cells [12]. However, transformed cells still possess the components of apoptotic mechanism, and it could be induced by various agents. The strategy of selectively killing tumor cells by inducing apoptosis could be used for cancer therapy [13,14], and the presented analysis provides information for the development of such strategy.

Apoptotic process is a complex sequence of signaling events and metabolic changes. The cascade of signaling events resulting in cell death is well studied. However, the signals to apoptosis could be seen as a result of severe distortions in metabolism. In this way, the metabolic changes could be primary events that activate or inhibit apoptotic process. For example, the stimulation of mitochondrial metabolism related to reactive oxygen species (ROS) production [15-17] or the inhibition of glycolisis [18] has been linked with activation of apoptotic cascade. Our objective was to understand whether relevant metabolic changes precede the development of apoptosis, or they just follow the progression of the apoptotic signaling program. To reveal the early metabolic changes, the metabolic effects of very low doses of edelfosine, which induce apoptosis in less than 5% of cellular population, were studied.

Synthetic antitumour ether phospholipid edelfosine (1-*O*-octadecyl-2-*O*-methyl*rac*-glycero-3-phosphocholine, ET-18-OCH_3_) selectively induces apoptosis in cancer cells [19-25]. The cell-killing mechanism of edelfosine is mediated by signalling events such as blocking some protein kinases [26] or activation of specific apoptotic receptors [21]. Also edelfosine induces the increase in mitochondrial reactive oxygen species (ROS) production [20,27], which could be a consequence of certain metabolic distortions. If metabolic changes are primary with respect to the development of apoptotic program, it could be expected that essential changes in cell metabolism could take place at low doses of such drug, which hardly induce apoptosis.

In order to find the metabolic changes caused by the low doses of edelfosine, Isodyn simulated the isotopomer distribution using the available enzyme kinetic information and the experimentally acquired ^13^C isotopomer distribution data. This approach allowed us to obtain sets of fluxes in the modeled metabolic network, which were consistent with the experimental distribution of mass isotopomers derived from labeled glucose in the presence of edelfosine. The determination of the metabolic conditions that promote apoptosis could be an essential contribution to the therapy of cancer.

## Results and Discussion

### Glucose consumption and lactate production

The rates of glucose consumption normalized per cell volume (for the convenience of comparison with the other intracellular fluxes) were defined taking into account the change of cell number and concentrations of medium glucose and lactate as it is described in "Methods". These fluxes are summarized in Table 1. These values calculated directly from experimental data were used in the fitting of isotopic isomer distribution described below.

**Table 1:** The metabolic fluxes of glucose consumption (J_Glc_) and lactate production (J_lac_).

	J_Glc _(mM/min)	J_Lac _(mM/min)
Con	0.09	0.1225
e0.5	0.1196	0.1565
e1	0.1359	0.1279

The fluxes are computed for the cells incubated in control conditions (con) and with 0.5 μg × mL^-1 ^or 1 μg × mL^-1 ^of edelfosine (e0.5 and e1 respectively).

### Measured isotopomer distributions

Since the objective of this work was to register metabolic changes that precede the development of apoptosis, very low doses of apoptosis inducing drug edelfosine were used (0.5 and 1 μg × mL^-1^) for ^13^C metabolite distribution experiments. After 48 hours of treatment, the dose of 0.5 μg × mL^-1 ^induced less than 1% of apoptosis whereas the dose of 1 μg × mL^-1 ^induced between 4 to 5% of apoptosis (measured as subG1 population with respect total number of cells). Incubation with [1,2-^13^C_2_]-glucose as a tracer resulted in a specific isotopomer (^13^C isotopic isomer) distributions in metabolites, which was measured by GC/MS techniques in medium lactate and ribose-5-phosphate (r5p) derived from intracellular RNA. The applied technique determined the fractions of different mass isotopomers: m0 (without any ^13^C labels), m1 (with one ^13^C label), m2 (with two ^13^C labels), etc. These fractions for control and edelfosine-treated cells are shown in Table 2.

**Table 2:** Isotopomer distribution in lactate secreted into the medium and RNA ribose.

Lactate:	control	sd	fit	0.5 μg/mL	sd	fit	1 μg/mL	sd	fit
m0	**0.7900**	0.003	0.7900	**0.7860**	0.001	0.7860	**0.7910**	0.003	0.7920
m1	**0.0097**	0.001	0.0096	**0.0099**	0.000	0.0099	**0.0098**	0.001	0.0098
m2	**0.1990**	0.003	0.1990	**0.2030**	0.001	0.2030	**0.1980**	0.003	0.1970
m3	**0.0013**	0.001	0.0013	**0.0013**	0.001	0.0013	**0.0010**	0.002	0.0011
Rib-5-P									
m0	**0.5480**	0.011	0.5480	**0.5570**	0.003	0.5570	**0.5790**	0.011	0.5790
m1	**0.2310**	0.004	0.2310	**0.2210**	0.007	0.2250	**0.2130**	0.004	0.2110
m2	**0.1490**	0.005	0.1490	**0.1410**	0.003	0.1430	**0.1340**	0.005	0.1390
m3	**0.0423**	0.001	0.0420	**0.0483**	0.005	0.0449	**0.0426**	0.001	0.0418
m4	**0.0299**	0.001	0.0296	**0.0325**	0.002	0.0293	**0.0312**	0.001	0.0291
dilution:			0.3450			0.2080			0.2920
χ			0.1760			4.7400			5.7500

The distribution was measured after 48 hours of incubation was assessed in Jurkat cells without drugs (control) or treated with either 0.5 μg × mL^-1 ^of edelfosine that caused less than 1% of apoptosis (0-1%) or with 1.0 μg × mL^-1 ^of edelfosine that caused 4-5% of apoptotis (4-5%). Best fit obtained with Isodyn is depicted in column "fit". For Ribose, dilution (initial fraction with respect to the newly synthesized fraction during the treatment) is also shown

### Simulation of measured isotopomer distributions

The distribution of isotopomer fractions of a metabolite contains information about the fluxes through the metabolic pathways of its formation. Roughly, when [1,2-^13^C_2_]-glucose is metabolized, anaerobic glycolysis produces mainly m2 lactate, whereas passage of labeled glucose through the TCA cycle (including anaplerotic pyruvate carboxylation, pyr→oaa) or the oxidative and non oxidative branches of the pentose phosphate pathway results in m1 and m3 lactate. Thus, the fractions of m1 and m3 with respect to m2 characterize the contribution of the TCA cycle and pentose phosphate pathway. The simulation and fitting the measured isotopomer distribution allows the determination of a set of metabolic fluxes, which correspond to the measured isotopomer distribution. The details of such determination are described in method section and in [8-10] and Additional file 1.

The results of data fitting by Isodyn are also presented in Table 2. The quality of fit is characterized by χ^2^, the sum of squares of differences between experimental and simulated data normalized by the standard deviations of experimental data. The ribose used for the analysis was extracted from cellular RNA. Thus, the isotopomer distribution in ribose contains information on both the label isotopomer distribution of the *de novo *synthesized nucleotides and also on the fraction of initial non-labeled nucleotides that were reused. The program calculates this initial fraction with respect to the one synthesized *de novo *during the treatment (as described in Additional file 1); it is referred in the tables as "dilution" and characterized RNA synthesis *de novo*. According to the data of Table 2, in edelfosine-treated cells dilution decreased, which indicates that a greater fraction of RNA was synthesized *de novo*.

### Analysis of metabolic flux profiles

Table 3 show the fluxes corresponding to the best fit shown in Table 2 and indicates the fluxes for which the difference between treated and non-treated cells are statistically significant. According to the table, to fit the measured isotopomer distribution in cell population where edelfosine induced ≤5% of apoptosis, glucose consumption (via hk, hexokinase activity) must increase, the TCA cycle (via pdh, pyruvate dehydrogenase activity) must be activated and pentose phosphate pathways must be inhibited with respect to the control.

**Table 3:** Simulated metabolic fluxes corresponding to the best fit presented in Table 2.

flux	Control		Edelf: 0.5 μg/mL		Edelf:1 μg/mL	
hk***	**0.0901118**	**1**	**0.12**	**1**	**0.135**	**1**
pfk	0.0968532	1.07481	0.120985	1.00821	0.135572	1.004237
fbpase	0.00716729	0.07954	1.46E-3	0.01219	0.0009715	0.007197
pep- > g3p	0.00694225	0.07704	0.00749499	0.06246	0.0025560	0.018933
g3p- > pep	0.186074	2.06492	0.246177	2.05148	0.271442	2.010681
pk	0.181224	2.01110	0.253755	2.11463	0.28068	2.079111
TCA- > pyr	2.23E-3	0.02470	0.0152166	0.12681	0.0118396	0.087701
lac- >	0.12219	1.35598	0.153661	1.28051	0.128901	0.954822
pdh***	**0.057984**	**0.64347**	**0.0815881**	**0.67990**	**0.140089**	**1.037696**
pc	1.05E-3	0.01165	0.0185053	0.15421	0.0116895	0.086589
CitSyn**	**0.0351159**	**0.38969**	**0.0547404**	**0.45617**	**0.120038**	**0.889170**
cit- > mal**	**0.0366362**	**0.40656**	**0.0549581**	**0.45798**	**0.120969**	**0.896067**
PPP*	**1.32E-4**	**1.47E-3**	**2.80E-6**	**2.33E-5**	**3.93E-6**	**2.91E-5**
r5p- >	4.38E-4	0.00486	6.84E-4	0.00570	0.0005799	0.004296
cit- > glt	3.50E-3	0.03884	2.13E-3	0.01775	0.0014090	0.010437
- > cit	0.00549634	0.06099	2.35E-3	0.01959	0.0023398	0.017332
Transketolase:						
xu5p- > s7p***	**0.00826654**	**0.09174**	**2.26E-7**	**1.88E-6**	**1.46E-6**	**1.08E-5**
s7p- > xu5p	0.0084719	0.09402	4.24E-4	0.00353	0.0003579	0.002651
f6p- > xu5p	5.66E-4	0.00628	3.54E-5	0.00030	3.03E-5	0.000224
xu5p- > f6p***	**5.38E-4**	**0.00597**	**3.45E-9**	**2.87E-8**	**1.89E-8**	**1.40E-7**
f6p- > s7p	0.00534401	0.05930	2.46E-4	0.00205	0.0001979	0.001466
s7p- > f6p	0.0052113	0.05783	4.49E-5	0.00037	3.08E-5	0.000228
xu5p < - > g3p***	**8.75E-4**	**9.71E-3**	**3.26E-8**	**2.71E-7**	**2.21E-7**	**1.64E-6**
f6p < - > e4p	3.48E-4	0.00386	3.75E-6	3.12E-5	2.63E-6	1.95E-5
r5p < - > s7p	0.0800302	0.88812	2.94E-3	0.02453	0.0023626	0.017501
Transaldolase:						
f6p- > s7p	1.54E-4	0.00171	2.57E-4	0.00214	0.0002003	0.001484
s7p- > f6p	2.48E-5	0.00028	2.25E-5	0.00019	4.54E-6	3.36E-5
f6p < - > g3p	2.64E-7	2.93E-6	1.09E-6	9.05E-6	2.51E-7	1.86E-6
s7p < - > e4p	0.0144573	0.16044	0.0053368	0.04447	0.0037384	0.027691

The fluxes were computed for cells incubated for 48 hours without drugs (control) or treated with either 0.5 μg × mL^-1 ^of edelfosine that caused less than 1% of apoptosis ( 0-1%) or with 1.0 μg × mL^-1 ^of edelfosine that caused 4-5% of apoptotis (4-5%). Left column for each condition shows absolute value of the flux (in mM × min^-1 ^in intracellular volume), right column shows the values normalized per respective glucose uptake (hk). The flux depicted as TCA- > pyr is a sum of fluxes through pepck and malic enzyme, that resulted in the same isotopomer distribution and contribute to the ^13^C enrichment in lactate from the TCA cycle. Arrows indicate the directions of fluxes; stars indicate the fluxes for which the absolute values (not normalized) are statistically different from control (***, p < 0.01; **, p < 0.05; *, p < 0.1).

Table 3 illustrates that a small change in the distribution of mass isotopomers shown in Table 2 could be a consequence of large changes in metabolic fluxes. Specifically, the flux through the TCA cycle (pdh, citrate synthase, flux from citrate to malate) increases almost three folds although the stimulation of apoptotic program can be measured in only 4-5% of cells. These fluxes normalized per respective glucose uptake are increased also, although not so tremendously. Thus, the low doses of edelfosine activate the whole central metabolism and even more activate the TCA cycle. In fact, it is not so simple to decide what value, normalized or not normalized, characterizes the TCA cycle activation better. Although glycolysis provides substrates for the TCA cycle, it is known that activation of glycolysis is not necessary coupled with the activation of the TCA cycle. For instance, in muscle cells starting active contractions, a hundred fold increase in glycolysis hardly activativates TCA cycle [28]. Glycolysis has much more capacity for activation, while the activation of the TCA cycle coupled directly with mitochondrial bioenergetics requires much more structural changes. If the TCA cycle is activated without the respective increase in the volume occupied by mitochondria, this activation probably could have negative consequences for cell survival.

Despite the changes in isotopomer distribution induced by a low dose of edelfosine is small, χ^2 ^criterion is sufficiently sensitive to them. The program fits the data for control with very small deviations (χ^2 ^= 0.18). Howener, if this "control" set of parameters is used to simulate the data for treated cells, χ^2 ^increases to 60, which indicates that the model well accepted as a simulator of metabolic fluxes in control cells becomes unacceptable for the edelfosine-treated cells. Subsequent fitting procedure decreases χ^2 ^to 5.75 (Table 2) just by increase of metabolic fluxes through the TCA cycle and changes in the pentose phosphate pathway as Table 3 indicates.

**Figure 1:**
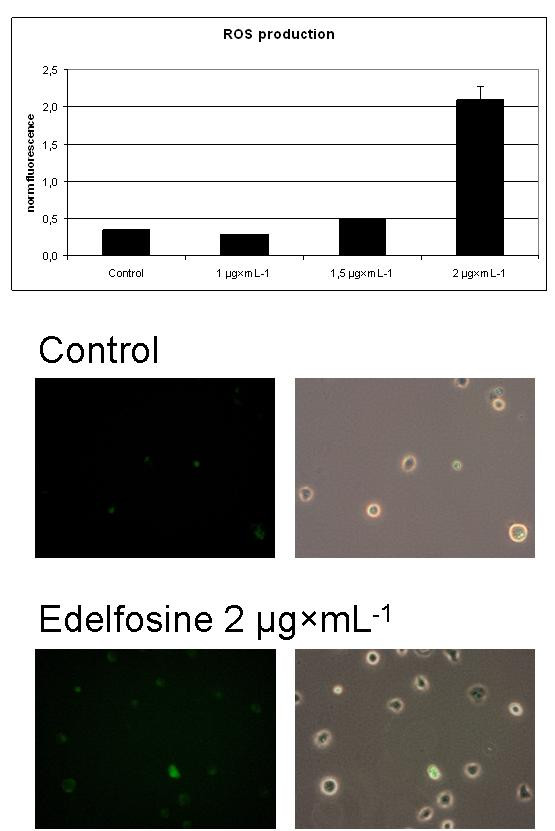
**Increase in DCF fluorescence in edefosine-treated Jurkat cells**. The DCF fluorescence as a result of ROS production was measured in a fluorescence microplate reader (top panel) and by fluorescence microscopy (left images, bottom two panels). Also shown are the phase-contrast images superimposed with the fluorescence images of the Jurkat cells (right images, bottom two panels).

The activation of the TCA cycle, revealed for a low dose of edelfosine, could be a reason for the activation of apoptotic process when higher doses of drug are applied. The main function of the TCA cycle in energy metabolism is to produce substrates for mitochondrial respiration. Therefore, the activation of TCA cycle favors the reduction of electron transporters. This is a factor that could switch the mitochondrial respiratory chain to the state of damaging generation of reactive oxygen species (ROS) [29], which is an important component of apoptotic process. The low doses used for the metabolomic studies did not increase ROS production, although their metabolic effect was significant. The increase of dose of edelfosine eventually produce significant increase of ROS, as it is shown in Figure 1. This observation, which is in line with other experimental studies reported elsewhere [20,27], validates the conclusion based on the isotopomer distribution data analysis.

Another important distinction in the revealed flux profiles of control and edelfosine-treated Jurkat cells is the difference in the fluxes of pentose phosphate pathway. In control cells all pentose phosphate fluxes were higher than those in edelfosine-treated cells (Table 3). Moreover, the model predicted an increase in ribose consumption for RNA synthesis in edelfosine-treated cells, which led to a decrease in ribose 5-phosphate concentration, such that its synthesis became practically unidirectional. Thus, newly synthesized ribose 5-phosphate was consumed for RNA synthesis to a greater extent in edelfosine-treated cells than control (see flux r5p, Table 3), in accordance with the value of ^13^C dilution in r5p (see Table 2). In contrast, in control cells, the newly synthesized ribose 5-phosphate is not massively used for RNA synthesis, but reutilized in the central energetic metabolism.

## Conclusions

The simulation of changes in isotopomer distribution induced by low doses of edelfosine revealed essential activation of the TCA cycle (including anaplerosis). Such direction of changes in metabolism could enhance ROS production when the higher doses of drug are applied. Indeed, when doses of edelfosine causing >5% of apoptosis rate were used (from 1 to 2 μg × mL^-1^) the increase of ROS production becomes measurable experimentally.

Another effect of the drug is the stimulation of RNA synthesis. Both effects of the drug are consistent: the increase of the TCA cycle fluxes are aimed in the increase of ATP energy production necessary for biosynthetic processes induced by such stress factor as edelfosine.

## Methods

### Structure of program and algorithms for isotopomer distribution analysis

The computer program "Isodyn", which we developed in C++, represents a simulation environment for the dynamics of metabolite labeling by ^13^C isotopes in metabolic reactions of living cells. For such simulations it uses a classical kinetic model of metabolic pathways linked with a module that computes the distribution of ^13^C isotopic isomers of metabolites. For the case of metabolic steady state it uses following algorithm for the simulation of dynamics of isotopomer distribution in metabolites.

1. To simulate reaching steady state in the kinetic model for total metabolite concentrations and fluxes for a given set of parameters.

2. To decompose the combined fluxes of kinetic model to the isotope-exchange fluxes, which differently affect isotopomer distribution.

3. To simulate the distribution of isotopomers using the total metabolite concentrations and decomposed fluxes obtained in steps 1 and 2.

Each simulation, performed through the steps 1-3, gives the distribution of isotopomers. The computed distribution is compared with the measured one using χ^2 ^criterion (see below) and a procedure of optimization is applied, which changes parameters and performs calculations each time passing through steps 1-3 with the objective to decrease χ^2^. The steps 1-3 and the procedure of optimization are described next.

#### Step 1

**Figure 2:**
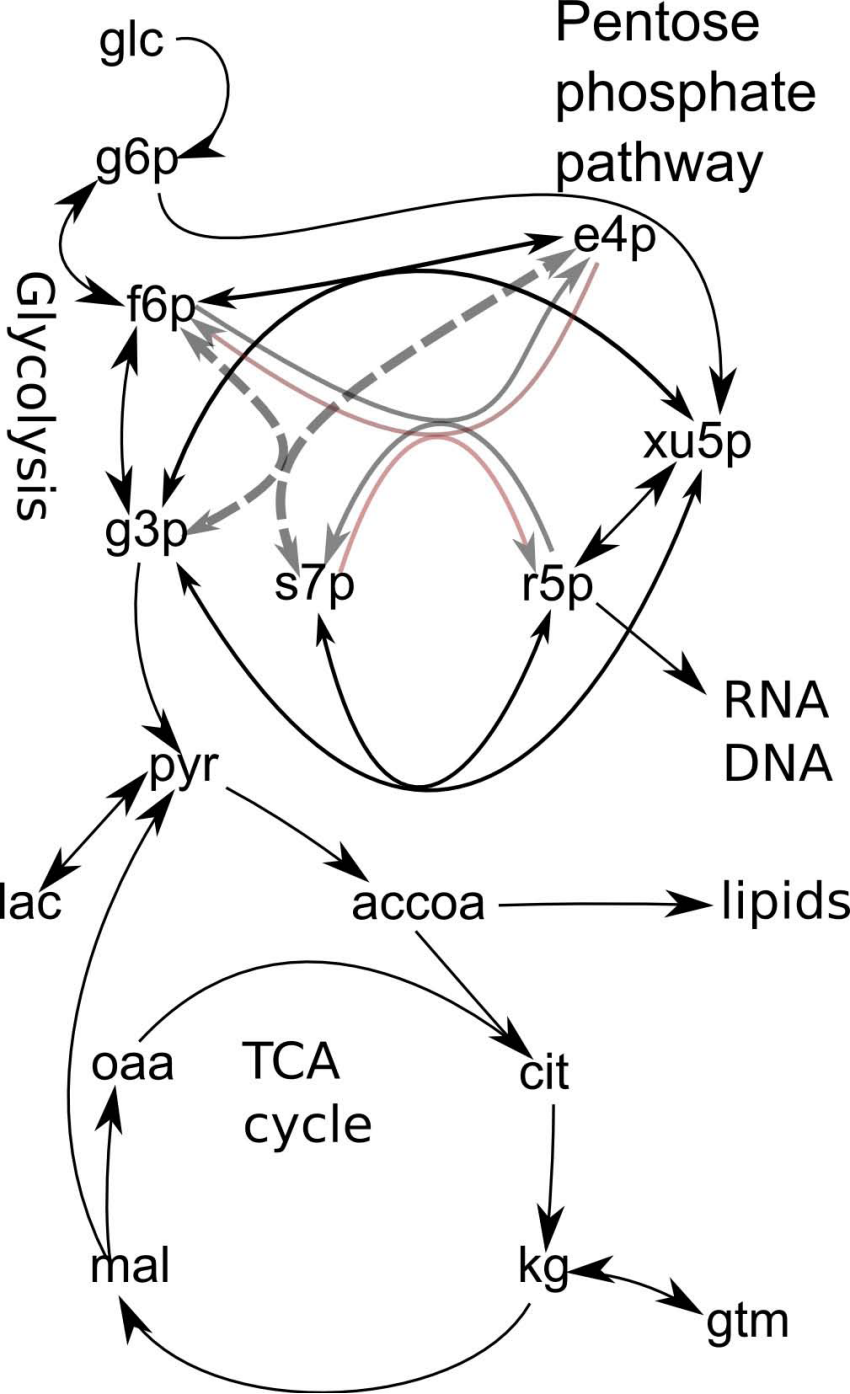
**Schematic representation of the main fluxes simulated in the model to study the edelfosine-induced apoptosis in the Jurkat cell line**. The description is presented in "Methods" and, in more detail, in Additional file 1.

The classical kinetic model is represented by a system of ordinary differential equations (ODE) simulating the evolution of concentrations of metabolites:

(1) dc/dt=N v(c,p)

here **c **= {c_1_, c_2_,..., c_m_} is the vector of m metabolite concentrations described by the model. The metabolites considered in this particular case are presented in Figure 2. This scheme reflects the simplifications accepted for the model, in particular, pentoses represented by r5p and xu5p, which are in fast equilibrium. Oxidative branch of pentose phosphate pathway is represented by one reaction converting g6p into pentose phosphates. One lumped reaction connects citrate with malate, g3p goes directly to pep. We described in detail the most important reactions where carbon skeleton changes and label is redistributed, and combined the reactions which either do not change the carbon skeleton or only release CO_2_.

**N **= {n_ij_}, i = 1,..., m, j = 1,..., k is stoichiometric matrix for m metabolites and k reactions and **v(c,p) **= {v_1_(**c,p**), v_2_(**c,p**),...,v_k_(**c,p**)}, is the vector of k reaction rates (metabolic fluxes), which are functions of concentrations and parameters such as Vmax. Concentrations vary with time in accordance with (1), and parameters are constant in each simulation, but they are unknown and could be found by fitting the experimental data. The rates for the most of enzymatic reactions are described by Michaelis' equation, although the reactions performing splitting/reformation of carbon skeleton of substrate molecule are considered in more detail taking into account the known elementary steps of reaction mechanism, as exemplified below for aldolase reaction. Matrix **N **is organized in such a way that production (input) and consumption (output) for the metabolites correspond to the arrows shown in Figure 2. Additional file 1 describes the whole set of differential equations of the model, which included the reactions of glycolysis, the TCA cycle, anaplerotic reactions, pentose phosphate pathway (PPP), exchange with extracellular glucose, lactate, and glutamate, and also the biosynthetic fluxes to nucleic acids and fatty acids. By solving these equations numerically, the program computes evolution of total metabolite concentrations and metabolic fluxes for a given set of parameters (Vmax, Km, rate constants).

#### Step 2

According to (1) differential equation for the concentration of a metabolite s (c_s_) is:

(2) $${\text{dc}}_{\text{s}}/\text{dt}=\Sigma {\text{n}}_{\text{sj}}{\text{v}}_{\text{j}}\left(\text{c},\text{p}\right)$$

The reactions j, which change concentrations c_s_, change also the concentrations of isotopomers **x_s _**= {x_s1_, x_s2_,...,x_sI_}. However, the net reaction rate v_i _accounted for kinetic model (1) could be composed of several isotope exchange processes, which differently affect isotopomer distribution and have to be accounted separately. Below we describe an example of such decomposition for aldolase reaction. Thus, reactions *v_i _*should be decomposed to several reactions u_i_: *v_i _*→ u_i1 _, u_i2 _.... This decomposition depends on reaction mechanism and is specific for each particular reaction. For instance, if a reaction does not produce any change in carbon skeleton of substrates, the decomposition implies only that the rates of transformation of substrate (*u_f_*) into product and reverse transformation of product into substrate (*u_r_*) must be calculated separately. The change of isotopic composition of reactants depends not only on net reaction rate (*v_i _*= *u_f _*- *u_r_*), but on the forward and reverse rates taken separately. If a reaction performs splitting/reformation of carbon skeleton of substrate molecule, additional isotope-exchange fluxes, different from forward and reverse reaction rates, could take place. Specifically, for aldolase reaction: fbp ↔ g3p + dhap, which proceeds through several elementary steps:

1. e + fbp ↔ e-fbp

2. e-fbp ↔ e-dhap + g3p

3. e-dhap ↔ e + dhap,

the fluxes through the whole reaction cycle (reactions 1-3) in the forward and reverse directions (*u_f_*, *u_r_*) and also the exchange flux of a half of fbp molecule with g3p (fbp ↔ g3p, steps 1 and 2 without entering to step 3, *u_e_*) should be evaluated. The latter does not change the total metabolite concentrations but it characterizes the interchange of first three atoms of fbp with g3p pool. *u_f _*and *u_r _*change also total concentrations, their values indicate the transition of last three atoms of f6p to and from dhap. The algorithms for the calculations of such isotope exchange fluxes for given parameters and metabolite concentrations are described elsewhere (Selivanov et al, 2005).

For transaldolase reaction, f6p+e4p ↔ s7p+g3p, in addition to the forward and reverse fluxes through the whole reaction cycle two additional isotope exchange fluxes should be evaluated: f6p ↔ g3p and s7p ↔ e4p.

For transketolase reactions (x5p+r5p ↔ s7p+g3p, f6p+g3p ↔ x5p+e4p, f6p+r5p ↔ s7p+e4p) in addition to the six forward and reverse fluxes through these three reaction cycles the isotope exchanges x5p ↔ g3p, f6p ↔ e4p and s7p ↔ r5p should be evaluated.

All the isotope exchange fluxes necessary for the calculation of isotopomer dynamics could be evaluated if the total metabolite concentrations are known after the execution of kinetic model. When the kinetic simulation is done, Isodyn calculates the necessary isotope exchange fluxes in one step, as explained in more details in (Selivanov et al, 2005) and Additional file 1.

#### Step 3

When all the preparations are done (i.e. total metabolite concentrations and necessary fluxes are computed), the system of equations of type (3) for all isotopomers could be solved. Isodyn calls a module, which performs the computation of isotopomer dynamics.

The differential equations for isotopomers of each of m considered metabolites after the decomposition of fluxes could be presented in the form similar to (2):

(3) $${\text{dx}}_{\text{si}}/\text{dt}=\Sigma_{\text{j}}\Sigma_{\text{h}}{\text{n}}_{\text{sj}}{\text{w}}_{\text{j}}\left({\text{u}}_{\text{jh}}\left(c,p\right),c,x\right)$$

here *w_j _*is individual reaction rate that changes the concentration of isotopomer *x_sj_*, which depends on the decomposition u_jh _of rate *v_j_*, vector of total concentrations **c **and isotopomer concentrations **x**.

Equation (3) describes the evolution of concentration of isotopomer i of substance s. To simulate the isotopomers distribution in metabolites, which are described by kinetic model (1), the equations of type (3) must be written for all the isotopomers of considered metabolites. This procedure is automated in our software.

To calculate the derivatives of isotopomers, the functions, which are specific for each reaction type, simulate the reaction mechanism in order to define the products for each isotopomer-substrate and also calculate the reaction rate for each given isotopomer transformation.

The simulation of reaction mechanism is based on operations with binary numbers. Since only two carbon isotopes are considered, any combination of carbons in the skeleton of a molecule could be reflected by a respective binary number, where "1" states for ^13^C and "0" states for ^12^C. In this way all hexose isotopomers could be represented by numbers from 000000 (decimal 0) to 111111 (binary equivalent of decimal 63), triose isotopomers range from 000 to 111 (decimal 7), etc.

When Isodyn simulates reactions for isotopomers it splits/recombines the binary representation of isotopomers in the same way as enzymes split and reform molecules. For instance since aldolase splits hexose (fbp) into two trioses, the respective Isodyn function splits binary numbers of fbp representatives, taking them one by one subsequently, e.g. when it takes "010101", it splits it to "010" and "101". The number 010101 (binary equivalent of decimal 21) indicates the position of the value of this isotopomer concentration (C_010101 _with binary index or C_21 _with decimal index) in the array of concentrations of fbp isotopomers. Knowing thus the position (and hence the concentration) of given isotopomer the algorithm calculates the reaction rate for a each isotopomer in a given reaction based on the total reaction rate (u^ald^_t_) and total concentration (C^fbp^_t_) known from the kinetic model simulation:

(4) $${\text{u}}^{\text{ald}}{}_{21}={\text{u}}^{\text{ald}}{}_{\text{t}}{\text{*C}}_{\text{21}}{\text{/C}}^{\text{fbp}}{}_{\text{t}}$$

this rate is subtracted from the derivative of 21^st ^isotopomer of fbp and added to the derivatives of trioses "010" (decimal 2) and "101" (decimal 5). The arrays of derivatives are organized in the same way as those for concentrations.

The same isotopomers could participate in various reactions. Isodyn simulates all of them adding the reaction rates (with correct sign) to the respective derivatives. The functions performing such simulations are described in Additional file 1. They constitute a library, which can be used selectively.

In general, large system for isotopomers (3) depends on and could be solved simultaneously with (1). However, if the dynamics of isotopomer distribution is simulated in the conditions of metabolic steady state, the procedure of numerical solution could be simplified so that the general kinetic equations (1) could be solved separately from the solution for isotopomers (3). This case is presented here as the steps 1-3.

Here is a small *example of application of above methodology*, where we organize the calculation of derivatives for all the isotopomers of system consisting of substrate and product of pdh reaction with constant pyruvate input: v_0 _→ pyr → accoa →. Let it is at metabolic steady state with initial isotopomer distribution for pyruvate (C_000_, C_001_, C_010_, C_011_, C_100_, C_101_, C_110_, C_111_) and accoa (C_00_, C_01_, C_10_, C_11_). Let v_0 _is constant input of uniformely labeled pyruvate (111). In this system at steady state all rates are v_0_, and let the computed total concentrations for some given set of parameters are C_pyr _and C_accoa_. Simulating this process Isodyn calls three functions that simulate respectively three reactions of the system. First function simulates constant input, it simply gives value v_0 _to the derivative of uniformely labeled pyruvate, not touching other derivatives (D_111 _= v_0_). Then, the function, which simulates pdh takes the arrays for pyr and accoa, calculates what accoa isotopomer is produced from each substrate simulating decarboxylation by removing the first digit from binary representation of pyr, calculates the rates for each isotopomer transition, and adds this rate to the value of respective derivative as it is demonstrated in Table 4.

**Table 4:** The algorithm for the automated calculation of reaction rates (column "rate") and time derivatives for all the isotopomers (shown in left column) of pyruvate (d_pyr _/dt) and accoa (d_accoa _/dt) exemplified for pdh reaction (pyr → accoa).

reaction	rate	d_pyr_/dt	d_accoa_/dt
000 → 00	u_000_= v_0_C_000_/C_pyr_	D_000_= -u_000_	D_00_= u_000_
001 → 01	u_001_= v_0_C_001_/C_pyr_	D_001_= -u_001_	D_01_= u_001_
010 → 10	u_010_= v_0_C_010_/C_pyr_	D_010_= -u_010_	D_10_= u_010_
011 → 11	u_011_= v_0_C_011_/C_pyr_	D_011_= -u_011_	D_11_= u_011_
100 → 00	u_100_= v_0_C_100_/C_pyr_	D_100_= -u_100_	D_00_= u_000_+u_100_
101 → 01	u_101_= v_0_C_101_/C_pyr_	D_101_= -u_101_	D_01_= u_101_+u_001_
110 → 10	u_110_= v_0_C_110_/C_pyr_	D_110_= -u_110_	D_10_= u_110_+u_010_
111 → 11	u_111_= v_0_C_111_/C_pyr_	D_111_= v_0_-u_111_	D_11_= u_111_+u_011_

Then Isodyn calls a function that simulates efflux of accoa as it is demonstrated in Table 5.

**Table 5:** The algorithm for the automated calculation of reaction rates (column "rate") and time derivatives for all the isotopomers (shown in left column) of accoa (d_accoa _/dt) exemplified for the reaction of efflux of accoa (accoa →).

reaction	rate	d_accoa _/dt
00 →	u_00_= v_0_C_00_/C_accoa_	D_00_= u_000_+u_100_-u_00_
01 →	u_01_= v_0_C_01_/C_accoa_	D_01_= u_101_+u_001_-u_01_
10 →	u_10_= v_0_C_10_/C_accoa_	D_10_= u_110_+u_010_-u_10_
11 →	u_11_= v_0_C_11_/C_accoa_	D_11_= u_111_+u_011_-u_11_

The pdh reaction (pyr → accoa) shown in Table 4 was already taken into account.

After the simulation in such a way of all the reactions of considered pathway, the whole array of derivatives (3) for all isotopomers at a given time point is formed. The function that calculates derivatives as described above could be called by any ODE solver, which solves the ODE system thus constructed. Isodyn implements several methods for ODE solving provided for C++ by Press et al [30], including fourth-order Runge-Kutta, Bulirsch-Stoer and Rosenbrock method for stiff systems. Also is implemented implicit Runge-Kutta 5^th ^order method for stiff systems (Radau5), described in [31] and backward differentiation formulas as their implemented in the solver DASSL [32] written in Fortran but linked with the C++ code of Isodyn. The implementation of various methods allows to find the fastest solution for a given problem. In the problem considered here the combination of DASSL for kinetic model with Runge-Kutta method as it is implemented in [30] for isotopomer distribution provided the fastest way to obtain solution.

The fact that solving the equations of kinetic model is followed by solving the system for isotopomers gives a way of checking solutions. Isodyn checks if the sum of isotopomers obtained after solving large isotopomer system is the same as total concentrations of metabolites obtained by kinetic model. The large isotopomer system could easily became stiff so that the employed method of solution does not provide necessary accuracy. In this case the program indicates the inaccuracy of solution.

#### Optimization

Simulation with an initial set of kinetic parameters gives a set of fluxes and corresponding isotopomer distribution, which could be compared with the measured distribution. Mass isotopomers of lactate and ribose were measured; to compare them with model prediction, the sums corresponding to respective measured mass isotopomers were calculated and the fractions with respect to the total amount were found. The difference between experimental data and the prediction was characterized by normalized square deviations (χ^2^=Σ_i_((f^e^_i_-f^t^_i_)/σ^e^_i_)^2 ^), where f^e^_i _is experimental mass isotopomer fraction, f^t^_i _is the predicted one, σ^e^_i _is experimental standard deviation. The objective was to find the set of parameters (Vmax for simple reactions described by Michaelis' equation with fixed Km, and rate constants for elementary steps of more complex reactions like transketoliase and transaldolase, described elsewhere [9], in total 29 parameters), which minimize χ^2^. To this end we subsequently used modified Simulated Annealing algorithm [33] and genetic algorithm. Random change of parameter values in Simulated Annealing we combined with Powell's method of coordinate descent [30], modifying each parameter in the direction that provides decrease of χ^2^. After each stochastic perturbation of parameters and descent to a local minimum of χ^2^, Isodyn saved the set of parameters and respective fluxes corresponding to the local minimum. These sets were used as an initial population for genetic algorithm. It performed crossover of these sets, "mutations" of randomly selected parameters and selection of obtained sets using the same criterion of χ^2^. After the finding the global minimum of χ^2 ^a range of fluxes corresponding to a specific level of confidence of χ^2^, was taken as a confidence interval corresponding to the chosen level of confidence [30].

### Experimental methods

#### Cell culture conditions

Jurkat (acute T cell leukaemia), obtained form the American Type Culture Collection, were grown in RPMI 1640 culture medium (Sigma-Aldrich Co, St Louis, MO) supplemented with 10% heat-inactivated FCS (PAA Laboratories, Pasching, Austria), 2 mM L-glutamine, 100 U × mL^-1 ^of penicillin and 100 μg × mL^-1 ^of streptomycin (Invitrogen, Paisley, UK) at 37°C in a humidified atmosphere of 5% CO_2_. At the time of treatment with edelfosine for isotopomeric distribution analysis, cells (250000 cells × mL^-1^) were incubated in 75cm^2 ^Petri dishes in the RPMI 1640 medium, and with 10 mmol × L^-1 ^of [1,2-^13^C_2_]-glucose (Sigma-Aldrich Co, St Louis, MO) at 50% isotope enrichment for 48 hours. At the end of the incubations, cells were centrifuged (1,500 rpm for 5 minutes) and medium was collected for glucose and lactate analysis, whereas cell pellets were frozen for RNA ribose analysis. Cells were counted with a haemocytometer, and edelfosine-induced apoptosis was assessed by flow cytometry using a fluorescence-activated cell sorter (FACS), as the percentage of cells in the sub-G0 region (hypodiploidy) in cell cycle analysis.

#### Glucose and lactate concentration

From culture medium, glucose and lactate concentration were determined as previously described [34,35] using a Cobas Mira Plus chemistry analyzer (HORIBA ABX, Montpellier, France) at the beginning and at the end of the treatments.

#### The calculation of fluxes of glucose consumption and lactate production normalized per intracellular volume

The glucose (C_g_) consumption rate (v) normalized per number of cells can be defined as follows. Glucose consumption rate for the given number of cells n_t _is

(5) $$\frac{dC_{g}}{dt}=v\times n_{t}$$

Assuming exponential cell growth n_t _could be expressed as:

(6) $$n_{t}=n_{0}\times e^{k\times t}$$

Separation of variables in (5) with substitution (6) gives:

(7) $$dC_{g}=\frac{v\times n_{0}}{k}\times e^{k\times t}d(k\times t)$$

Integration of (7) gives

(8) $$\Delta C_{g}=\frac{v}{k}(n_{t}-n_{0})\;\text{and}\;v=\frac{\Delta C_{g}\times k}{n_{t}-n_{0}}$$

k could be defined from (6):

$k=\frac{\ln(n_{t}/n_{0})}{t}$, and substitution in (8) gives:

(9) $$v=\frac{\Delta C_{g}\times \ln(n_{t}/n_{0})}{(n_{t}-n_{0})\times t}$$

During the 48 hours of incubation without edelfosine (control) the number of cells in average increased from 0.25 × 10^6 ^cells × mL^-1 ^to 0.36 × 10^6 ^cells × mL^-1^. Glucose concentration decreased in average from 8.66 mM to 4.76 mM.

(10) $$v=\frac{3.9\mu mol\times \ln(0.36/0.25)}{10^{6}\times (0.36-0.25)cells\times 2880\min}=0.0045\frac{\mu mol}{10^{6}cells\times \min}$$

This consumption of glucose induces changes in intracellular concentrations of metabolites. To compare this flux with intracellular metabolic fluxes the units were changed to mM/min in the intracellular volume by dividing the above value of v to the volume of 10^6 ^cells, which was 0.05 mL:

(11) $$v=0.0045\frac{\mu mol}{10^{6}cells\times \min}=0.09\frac{mM}{\min}$$

This value characterizes the change of intracellular concentration per minute and all the fluxes were expressed in the same units.

#### Lactate isotopomer distribution

Lactate from the cell culture medium was extracted by ethyl acetate after acidification with HCl. Lactate was derivatized to its propylamideheptafluorobutyric form and the m/z 328 (carbons 1-3 of lactate, chemical ionization) was monitored as described [36].

#### RNA Ribose isotopomer distribution

RNA ribose was isolated by acid hydrolysis of cellular RNA after Trizol (Invitrogen) purification of cell extracts. Ribose isolated from RNA was derivatized to its aldonitrile acetate form using hydroxyl-amine in pyridine and acetic anhydride. We monitored the ion cluster around the m/z 256 (carbons 1-5 of ribose, chemical ionization) to find the molar enrichment and distribution of ^13^C labels in ribose^43^.

#### Gas Chromatography/Mass Spectrometry

Mass spectral data were obtained on the HP5973 mass selective detector connected to an HP6890 gas chromatograph. The settings are as follows: GC inlet 230°C, transfer line 280°C, MS source 230°C, MS quad 150°C. An HP-5 capillary column (30 m length, 250 μm diameter, 0.25 μm film thickness) was used for analysis of ribose and lactate.

*In vitro *experiments were carried out using duplicate cultures each time for each treatment regimen. Mass spectral analyses were carried out by three independent automated injections of 1 μl of each sample and were accepted only if the standard sample deviation was less than 1% of the normalized peak intensity.

#### ROS production

ROS production was monitored using the fluorescente probe 2',7'-dichlordihydrofluorescein diacetate (H_2_DCFDA) (Invitrogen, Carlsbad, CA). Jurkat cells (150,000 cells/well) were grown in RPMI 1640 medium (as described above) in 6-well culture plates. Prior to Edelfosine treatment, cells were harvested by centrifugation and preloaded with 10 μM H_2_DCFDA in PBS for 30 min at 37°C, followed by wash in PBS to remove unloaded probe, addition of fresh medium containing 0-2 μg × mL^-1 ^edelfosine, and incubation at 37°C/5% CO_2 _for 48 hr. After treatment, cells were harvested, washed in PBS twice, and resuspended in PBS before DCF fluorescence was read with excitation and emission wavelengths at 495 nm and 527 nm, respectively. All fuorescence readings were normalizad to cell counts. The same treatment was also performed for cells grown in cover slips and DCF fluorescence examined using a BX51 fluorescence microscope (Olympus, Melvilla, NY).

## List of abbreviations

Metabolites: glc: glucose; g6p, glucose-6-phosphate; f6p: fructose-6-phosphate; g3p: glyceraldehydes-3-phosphate; dhap: dihydroxyacetone phosphate; pep: phosphoenolpyruvate; pyr: pyruvate; lac: lactate; xu5p: xylulose-5-phosphate; r5p: ribose-5-phosphate; s7p: sedoheptulose-7-phosphate; e4p: erythrose-4-phosphate; accoa: acetyl-CoA; cit: citrate; kg: α-ketoglutarate; glut: glutamate; oaa: oxaloacetate. Enzyme reactions: hk: hexokinase; pfk: phosphofructokinase; fbpase: fructose bisphosphatase; pk: pyruvate kinase; pepck: phosphoenolpyruvate caboxykinase; pdh: pyruvate dehydrogenase complex; pc: pyruvate carboxylase; CitSyn: citrate synthase.

## Authors' contributions

VAS performed the theoretical analysis of data and wrote the paper, PV performed experiments and wrote the paper, FM analyzed data, TWMF analysed data and performed experiments, WNPL analysed data, MC analysed data and wrote the paper. All authors read and approved the final manuscript.

## Supplementary Material

Additional file 1 **Supplementary materials**. PDF containing supplemental figures and other information. Click here for file
