# Supplementary material for: Edelfosine-induced metabolic changes in cancer cells that precede the overproduction of reactive oxygen species and apoptosis
Source: BMC Syst Biol. 2010 Oct 6;4:135. doi: 10.1186/1752-0509-4-135 (PMC2984393; doi:10.1186/1752-0509-4-135)
Supplement: Additional file 1 — Supplementary materials. PDF containing supplemental figures and other information. [file 1752-0509-4-135-S1.PDF]

Classical kinetic model of central metabolism

Figure 1 illustrates an example of kinetic model of central metabolism, which is considered here.

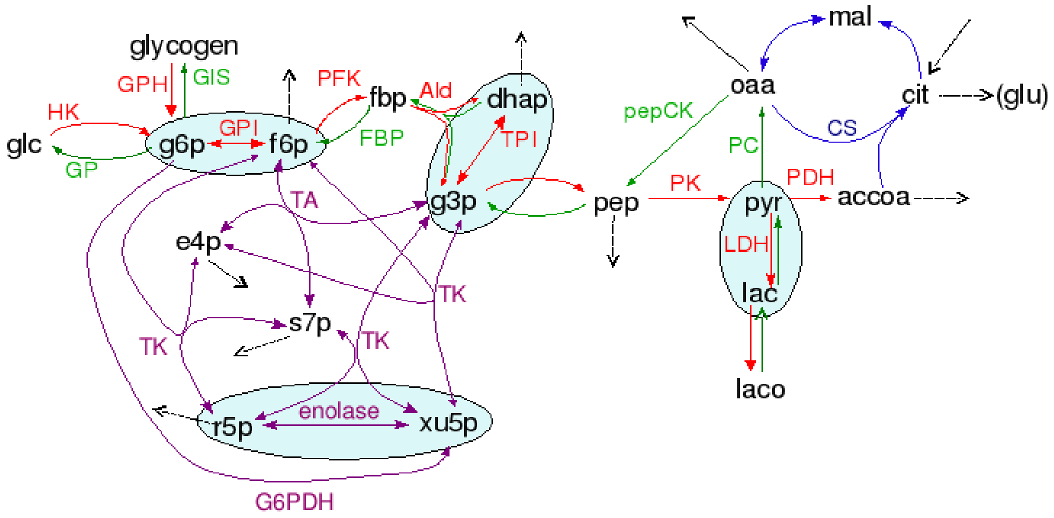

Figure 1. Scheme of a kinetic model of central intracellular metabolism. Red arrows indicate glycolysis, magenta - pentose-phosphate pathway, green - gluconeogenesis, and blue - tricaboxylic acid (TCA) cycle. Black arrows indicate exchanges with metabolites, external with respect to the model variables: glc (glucose), g6p (glucose 6-phosphate), f6p (fructose 6-phosphate), fbp (fructose phosphate), g3p (glyceraldehyde 3-phosphate), dhap (dihydroxyacetone phosphate), pep (phosphoenol pyruvate), pyr (pyruvate), lac (lactate intracellular), laco (lactate extracellular), accoa (acetyl coenzyme A), oaa (oxaloacetate), cit (citrate), mal (malate), oaa (oxaloacetate), e4p (eritrose 4-phosphate), s7p (sedoheptulose 7-phosphate), r5p (ribose 5-phosphate), xu5p (xylulose 5-phosphate), and glu (glutamate). These variables are substrates and products of HK (hexokinase), GP (glucose 6-phosphatase), GPH (glycogen phosphorylase), GPI (glucose phosphate isomerase), PFK (phosphofructokinase), FBP (fructose bisphosphatase), Ald (aldolase), TPI (triose phosphate isomerase), pepCK (pep carboxykinase), PK (pyruvate kinase), LDH (lactate dehydrogenase), PDH (pyruvate dehydrogenase complex), CS (citrate synthase), TA (transaldolase), TK (transketolase), and enolase. The reactions between g3p and pep, cit and mal, mal and oaa are lumped and described in the model as one reaction step. g3p and dhap, g6p and f6p, pyr and lac, r5p and xu5p are assumed to be in fast equilibrium and constitute united pool.

The following system of differential equations describes the evolution of variables:

h6p' =vHK + vFBP - vPFK - vPPP - vTK2 - vTA - vGIS- vf6o;

t3p' =2*vAld - vg3pep + vTK + vTA - vdho;
fbp' = vPFK - vFBP - vAld;
npep' = vg3pep + vpepCK - vPK - vpepo;
lp' = vPK - vPC - vPDH - vlaco;

$accoa' = v_{PDH} - v_{CS} - v_{coao};$

$oaa' = v_{PC} + v_{maloa} + v_{citin} - v_{CS} - v_{pepCK} - v_{oao};$ (1)

$cit' = v_{CS} - v_{citmal} - v_{citglu};$

$glgn' = v_{GlS} - v_{GPH};$

$p5p' = v_{G6PDH} - v_{TK} + v_{TK1} - v_{r5o};$

$e4p' = v_{TK2} - v_{TA} - v_{e4o};$

$s7p' = - v_{TK1} + v_{TA} - v_{s7o};$

$mal' = v_{citmal} - v_{maloa};$

Here h6p is g6p + f6p, which are in fast equilibrium; similarly t3p = g3p+dhap, lp = lac + pyr, and p5p = r5p + xu5p. v with subscripts are the reaction rates for respective enzymes as indicated in Figure 1. Their expressions are:

$v_{HK} = V_{HK},$

$v_{PFK} = V_{PFK}*[f6p]/(K_{f6p} + [f6p]);$

$v_{FBP} = V_{FBP}*[fbp]/(K_{fbp} + [fbp]);$

$v_{g3pep} = V_{g3pep} *[g3p]/(K_{g3p} + [g3p]) - V_{pepg3} *[pep]/(K_{pep} + [pep]);$

$v_{PK} = V_{PK} *[pep]/(K1_{pep} + [pep]);$

$v_{pepCK} = V_{pepCK} *[oaa]/(K_{oaa} + [oaa]);$

$v_{laco} = V_{lacf} *[lac]/(K_{lac} +[lac]) - V_{lacf} *[laco]/(K_{laco} +[laco]);$ (2)

$v_{PDH} = V_{PDH} *[pyr]/(K_{pyr} + [pyr])*(K1_{oaa}+ [oaa])/K1_{oaa};$

$v_{PC} = V_{PC} *[pyr]/(K1_{pyr} + [pyr])*(K1_{coa}+ [accoa])/K1_{coa};$

$v_{CS} = V_{CS} *[oaa]*[accoa]/(K2_{oaa} + [oaa])/(K_{coa} + [accoa])$

$v_{GlS} = V_{GlS};$

$v_{citmal} = V_{citmal} *[cit]/(K_{cit} + [cit]);$

$v_{G6PDH} = V_{G6PDH}*[g6p]/(K_{g6p} + [g6p]);$

$v_{maloa} = V_{maloa}*[mal]/(K_{mal} + [mal]) - V_{oamal}*[oaa]/(K_{oaa} + [oaa]);$

The effluxes of metabolites v_{f6o}, v_{dho}, v_{pepo}, v_{coao}, v_{oao}, v_{r5o}, v_{e4o}, v_{s7o} were described as Michaelis' functions of respective substrates. The reactions performed by aldolase, transaldolase and transketolase are treated specifically, because they catalyse, in fact, several isotope-exchange fluxes, which have to be taken into account for the correct connection with the module for simulation of isotopomer distribution.

For aldolase reaction the mechanism indicated in Figure 2 was taken into account (Mulquiney and Kuchel, 1999):

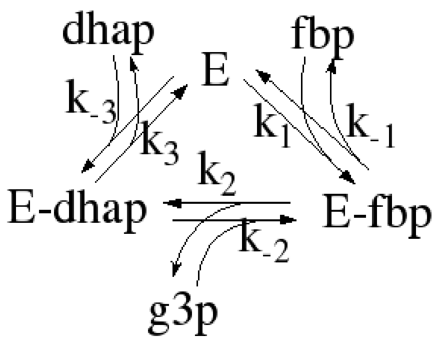

Figure 2. Kinetic scheme of aldolase reaction. The enzyme reversibly binds fbp, splits it and releases g3p and then dhap. k_i and k_{-i} are the rate constants of forward and reverse elementary reactions.

Reaction rate in this scheme is the difference between forward and reverse elementary reactions, which is the same for all steps. For instance it could be expressed as

$$v_{Ald} = k_1*[E]*[fbp] - k_{-1}*[E\text{-}fbp] \quad (3)$$

The enzyme forms could be expressed through the elementary rate constants, using for instance King-Altman algorithm:

$$v_{Ald} = E_0*(k_2*k_3*k_1*fbp - k_{-3}*dhap*k_{-2}*g3p*k_{-1})/(k_{-1}*g3p*k_{-2} + k_2*k_3 + k_3*k_{-1} + k_3*k_1*fbp + k_{-3}*dhap*k_{-2}*g3p + k_1*fbp*k_{-2}*g3p + k_1*fbp*k_2 + k_{-1}*k_{-3}*dhap + k_{-3}*dhap*k_2), \quad (4)$$

where E_0 is the total amount of enzyme.

For transaldolase reaction the mechanism indicated in Figure 3 was taken into account (McIntyre et al, 1989):

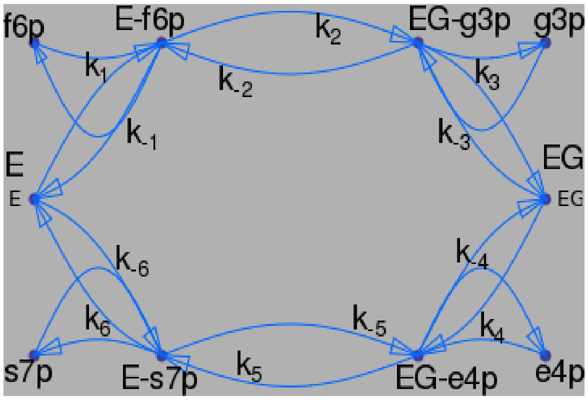

Figure 3. Kinetic scheme of transaldolase reaction. The enzyme reversibly binds fructose 6-phosphate, splits it and releases glyceraldehyde 3-phosphate, keeping three-carbon fragment bound to the active site. Then it binds eritrose 4-phosphate, facilitating the formation of covalent bond with 3-carbon fragment from fructose 6-phosphate and releases sedoheptulose 7-phosphate.

Reaction rate in this case is also expressed as a difference between forward and reverse elementary reactions of any of the six reversible steps.

v_{TA}=k_1*[E]*[f6p] - k_{-1}*[E-f6p] (5)

It was also expressed through the elementary steps, similar to (4).

Transketolase (McIntyre et al, 1989) catalyses three reactions and the respective scheme, which accounts for the competition of substrates for the same enzyme is shown in Figure 4:

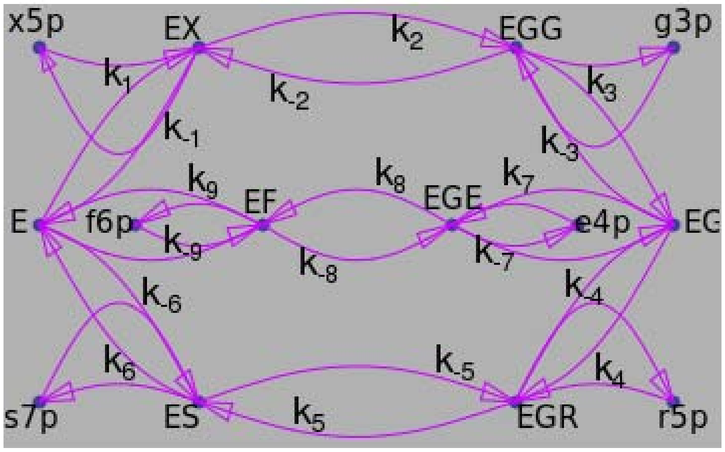

Figure 4. Kinetic scheme of transketolase reaction. The enzyme reversibly binds xylulose 5-phosphate, fructose 6-phosphate, or sedoheptulose 7-phosphate, splits the bound substrate and releases respectively glyceraldehyde 3-phosphate, erythrose 4-phosphate, or ribose 5-phosphate, keeping two-carbon fragment bound to the active site.

According to the scheme, the rate of consumption of xu5p and production of g3p could be expressed as

v_{TK1}= k_1*[E]*[xu5p] - k_{01}*[E-xu5p] (6)

The rate of consumption of f6p and production of e4p could be expressed as

v_{TK2}= k_7*[E]*[f6p] - k_{07}*[E-f6p] (7)

The rate of consumption of s7p and production of r5p could be expressed as

v_{TK3}= k_{06}*[E]*[s7p] - k_6*[E-s7p] (8)

All the reaction rates for transketolase and for transaldolase were expressed through the elementary reaction rate constants, similar to that shown for aldolase reaction (4), although the expressions were much longer. Deriving these expressions we have taken into consideration the known equilibrium constants for the reactions. This allowed to exclude one unknown parameter for each reaction. The values of parameters were defined by fitting the distribution of isotopomers measured experimentally after the incubation of living cells in the medium containing ^{13}C-glucose. The best fit values are presented below as the result of fitting.

Interface between the classical kinetic model and computing isotopomer distribution.

The model presented above can be used to compute the time course of metabolic fluxes and metabolite concentrations, which correspond to a specific set of parameters. Normally, the objective of analysis with kinetic model is to define the set of parameters (and, consequently, the characteristics of system operation and regulation), which most precisely simulate the behavior of real biological systems. However, usually only few products of intracellular metabolism are available for measurement and the lack of information highly restricts the results of kinetic model application. The distribution of labeled isotopomers even in the same metabolites, which is available for measurement by mass spectrometry or NMR, provide much more information about the system operation, which allows to define unambiguously much more characteristics of the biological system in situ. To be able to analyze the measured isotopomer distribution, the model must be modified as specified next.

For the calculation of metabolite concentration change, only the values of derivatives (1) are necessary and they are computed according to the expressions for reaction rates (2 - 8). However, isotope exchange could proceed even without a change in metabolite concentrations, therefore to simulate isotopomer distribution, the isotope exchange fluxes must be accounted for separately. Such isotope exchange fluxes are:

- for practically irreversible reactions, the reaction rates calculated using respective equations from (2) coincide with total isotope-exchange flux. Examples of such reactions in (2) are v_{HK}, v_{PFK}, v_{FBP}.
- for reversible bidirectional reactions, which proceed without splitting and reformation of carbon skeleton, the forward and reverse components of the net rate must be taken into account separately. For instance if a net reaction rate is described as v=vf-vr, where vf and vr are the forward and reverse components of the net rate, the values of components vf and vr make separate contribution in the isotopomer distribution and therefore their values must be stored separately and then used for isotopomer distribution simulation described below. Examples of such reactions in (2) are v_{g3pep}, v_{laco}, v_{maloa}.
- for reversible reactions, in which splitting and reformation of carbon skeleton take place, normally there exist specific isotope-exchange fluxes that do not affect a metabolite concentration as a whole, but change its isotopomer content. In the model we have implemented three enzymes performing such reactions, aldolase, transaldolase and transketolase. Now we consider them in details.

Aldolase

The scheme present in Figure 5 shows the possible isotope-exchange fluxes in aldolase reaction:

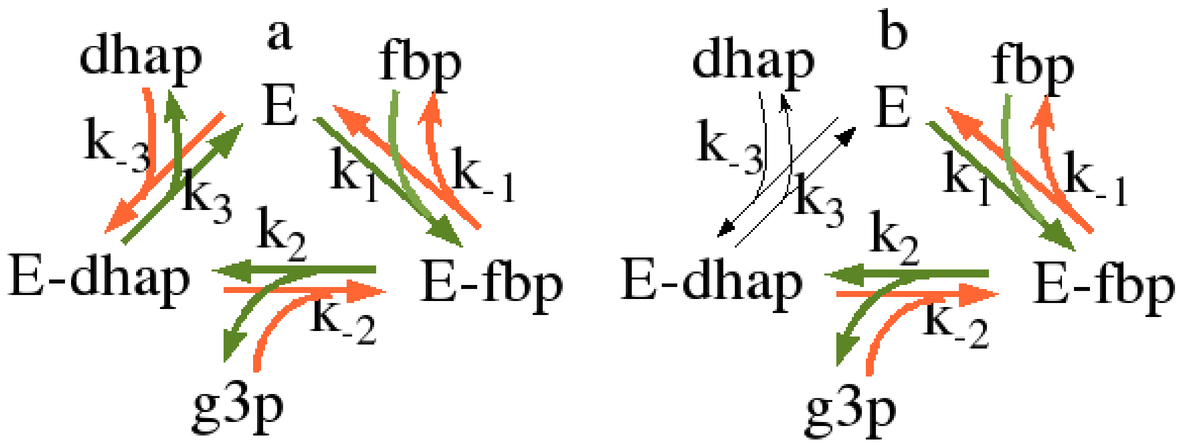

Figure 5. Various isotope-exchange fluxes in aldolase-catalysed reaction. **(a)**, forward and reverse reaction fluxes, which, respectively, transform the whole molecule of fructose bisphosphate in two trioses released into triose pool (green lines), and vice versa, two trioses in fructose bisphosphate (orange lines). **(b)** partial fluxes resulted in the interchange of only half a molecule of fructose with the pool of trioses.

The flux, shown in Figure 5a with green lines, transforms the whole molecule of fbp in the pool of trioses. It constitutes only a part of the flux v_3 (with rate constant k_3), because a part of v_3 produces dhap originated not from fbp, but from the same pool trioses, bound to the enzyme through the reaction v_{-3}. The steady state fraction of v_3, which produces dhap originated from fbp (or fraction of bound dhap originated from fbp (P^f_{ed})) can be expressed as the ratio of input of molecules originated from fbp to total input in E-dhap:

$$P^f_{ed} = v_2 * P^f_{ef} / (v_2 + v_{-3}) \quad (9)$$

Here P^f_{ef} is a fraction of v_2, which brings carbons originated from fbp to E-ghap[1] (or fraction of bound fbp originated from fbp). P^f_{ef}, in turn, can be expressed as the ratio of input of molecules originated from fbp to total input in E-fbp:

$$P^f_{ef} = (P^f_{ed} * v_{-2} + v_1)/(v_1 + v_{-2}) \quad (10)$$

The solution of equations (9) and (10) is

$$P^f_{ed} = v_2 * v_1 / (v_2 * v_1 + v_{-3} * v_1 + v_{-3} * v_{-2}) \quad (11)$$

where the rates v_i could be expressed through the rate constants and substrate concentrations similar to (4). The forward flux through the whole cycle (marked green in Figure 5a is

$$vf = v3 * P^f_{ed} = v_1 * v_2 * v_3 / (v_2 * v_1 + v_{-3} * v_1 + v_{-3} * v_{-2}) \quad (12)$$

Similarly, the reverse flux of fbp formation (marked orange in Figure 5a) from triose phosphates (P^t_{ef}) is a fraction of v_{-1}, which brings atoms originated in triose phosphates. It could be described similar to (9).

$$P^t_{ef} = v_{-2} * P^t_{ed} / (v_1 + v_{-2}) \quad (13)$$

---

1 Another fraction of v_2, $(1-P^f_{ef})$ brings carbons originated from triose pool, which were bound through reaction v_{-3} and transferred to E-fbp through reaction v_{-2}

Here $P^t{}_{ed}$ is a fraction of v_{-2}, which brings carbons originated from triose phosphates to E-fbp (or fraction of bound dhap originated from triose phosphate pool). $P^t{}_{ed}$, in turn, can be expressed as the ratio of input of molecules originated from triose phosphates to total input in E-fbp:

$$P^t{}_{ed} = (v_{-3} + v_2 * P^t{}_{ef})/(v_2 + v_{-3}) \quad (14)$$

Solving (13) and (14) gives the expressions for and through the elementary reaction rates, which could be easily expressed through the rate constants and substrate concentrations. The reverse flux (marked green in Figure 5a) is expressed similarly to (12):

$$v_r = v\text{-}1 * P^t{}_{ef} = v_{-1} * v_{-2} * v_{-3}/(v_2 * v_1 + v_{-3} * v_1 + v_{-3} * v_{-2}) \quad (15)$$

Figure 5b shows two additional fluxes, which only exchange half a molecule of fbp with triose phosphate pool. Like in the cases described above, the fraction of last tree carbons originated from fbp ($P^{fg}{}_{ed}$) in E-fbp is

$$P^{fg}{}_{ef} = v_1/(v_1 + v_{-2}) \quad (16)$$

and the forward (green in Figure 5b) flux:

$$v_{fg} = v_1 * v_2/(v_1 + v_{-2}) \quad (17)$$

The fraction of last three carbons originated from fbp ($P^{fg}{}_{ed}$) in E-fbp is

$$P^{tg}{}_{ef} = v_{-2}/(v_1 + v_{-2}) \quad (18)$$

and the reverse (orange in Figure 5b) flux:

$$v_{rg} = v_{-1} * v_{-2}/(v_1 + v_{-2}) \quad (19)$$

The forward partial flux (17) contains accounts also for the flux (12) through the whole reaction cycle, as well as the reverse partial flux (19) accounts for the reverse flux (15) through the whole reaction cycle. The "pure" isotope exchange flux, which does not change metabolite concentration, is the difference between partial and whole flux of the same direction. This difference is the same for the forward and reverse directions (because it is the same flux passing through the forward and reverse elementary reactions of the same steps).

$$v_e = v_{fg} - v_f = v_{rg} - v_r; \quad (20)$$

Thus, the model accounts for three isotope-exchange fluxes related with aldolase activity: forward and reverse flux through the whole cycle of enzyme reaction, and pure isotope exchange flux between f6p and g3p without change of total concentrations of these metabolites. They are not used in classical kinetic simulations, where only the net flux is important, but they are necessary for the subsequent simulation of isotopomer distribution.

Transaldolase

Transaldolase catalyzes splitting a substrate-donor, fructose 6-phosphate, between third and fourth carbon atoms releasing the last part of f6p molecule as glyceraldehyde 3-phosphate. If eritrose 4-phosphate is used as a substrate-acceptor, it could be covalently bound to the three-carbon fragment remaining in the active site and thus formed sedoheptulose 7-phosphate is released. This reaction mechanism is illustrated in Figure 6, which shows that four different isotope-exchange fluxes are related with transaldolase reaction.

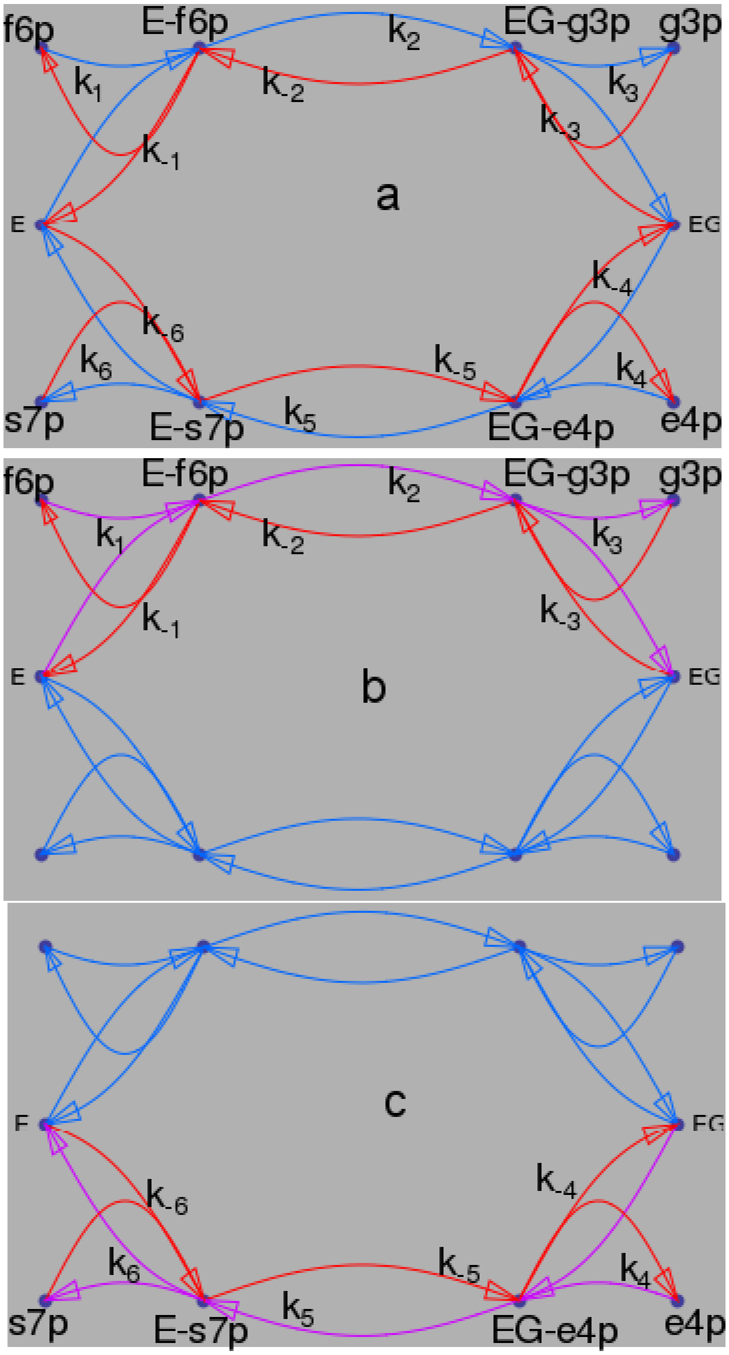

Figure 6. Isotope-exchange reactions catalysed by transaldolase. **(a)** shows forward (blue) and reverse (red) fluxes through the whole reaction cycle, which exchange three first carbons

between f6p and s7p. This exchange is also coupled is also coupled with exchange of three last carbons of f6p and g3p, and four last carbons of s7p and e4p. **(b)** shows forward (magenta) and reverse (red) half-reactions, which complete the cycle that only exchange three last carbons of f6p and g3p without a change of total metabolite concentrations. **(c)** shows forward (magenta) and reverse (red) half-reactions, which complete the cycle that only exchanges four last carbons of s7p and e4p.

Two fluxes through the whole cycle (Figure 6a), forward and reverse, exchange first three carbons between f6p and s7p coupled with exchange between f6p and g3p, and also between s7p and e4p.

F6p → E-f6p→EG-g3p→EG→EG-e4p→E-s7p → s7p

f6p ← E-f6p←EG-g3p←EG←EG-e4p←E-s7p←s7p

Moreover, just half-reactions also exchange isotopes between f6p and g3p, or between s7p and e4p, as Figures 6b and 6c indicate.
f6p + E ↔ E-f6p ↔ EG-g3p ↔ EG + g3p
s7p + E ↔ E-s7p ↔ EG-e4p ↔ EG + e4p

The expressions for all isotope exchange fluxes through the elementary rate constants and substrate concentrations were obtained by constructing and solving the equations, describing the fractions of elementary reaction rates transferring the carbons of specific origin. Such equations are similar to (9), (10), (13), (14), (16), and (18) for aldolase, but the expressions for fluxes are larger, since the reaction mechanism is more complicated.

Transketolase

Transketolase catalyzes splitting a substrate-donor, ketose phosphate, between second and third carbon atoms and releases the last part of molecule as aldose phosphate. Another aldose could be covalently bound to the two-carbon fragment remaining in the active site thus forming new ketose phosphate. The various isotope-exchange fluxes related with transketolase reaction, are illustrated in Figure 7.

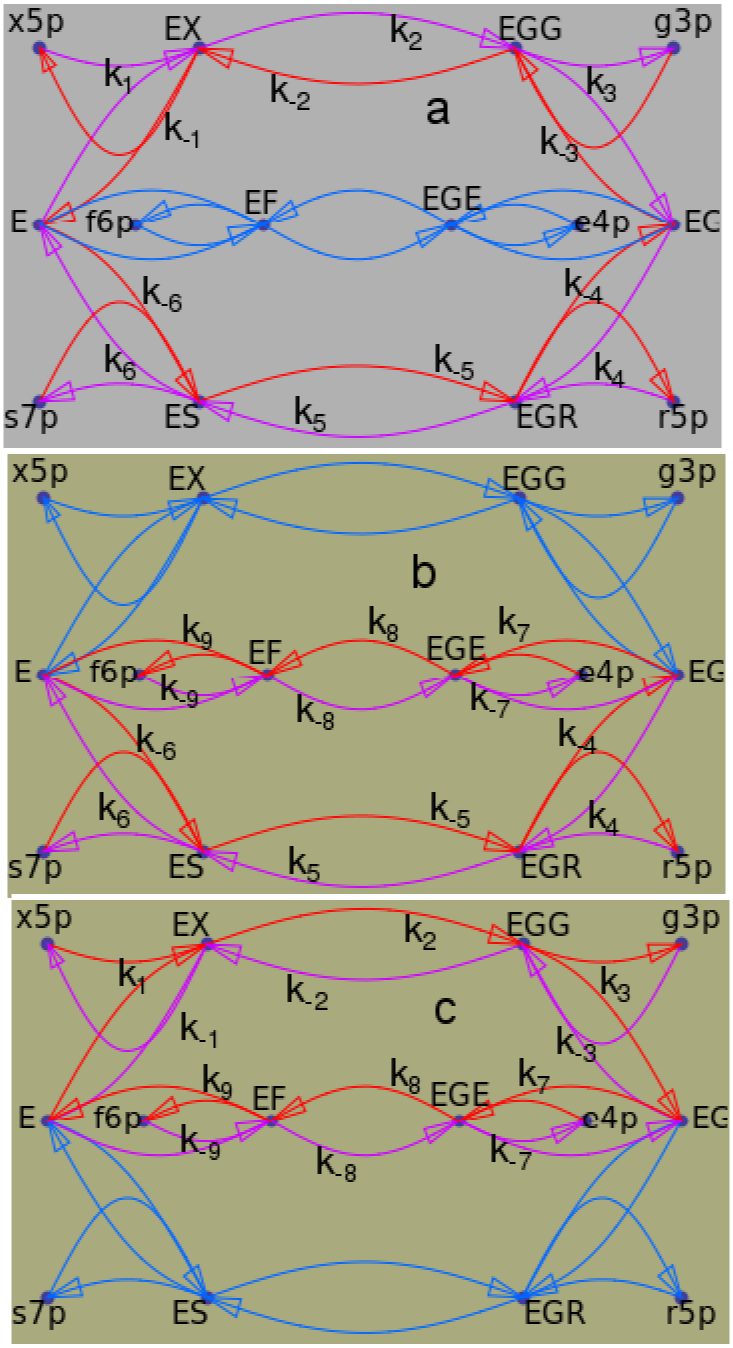

Figure 7. Isotope-exchange in three reactions catalysed by transketolase competing for the same enzyme. **(a)** shows forward (magenta) and reverse (red) fluxes through the whole cycle of reaction xu5p + r5p ↔ g3p + s7p, which exchange two first carbons between xu5p and s7p. This exchange is also coupled with exchange of three last carbons of xu5p and g3p, and five last carbons of s7p and r5p. **(b)** shows forward (magenta) and reverse (red) fluxes through the whole cycle of the reaction f6p + r5p ↔ e4p + s7p, which exchange two first carbons of f6p and s7p. This exchange is also coupled with exchange of four last carbons of f6p and e4p, and five last carbons of s7p and r5p. **(c)** shows forward (magenta) and reverse (red) fluxes through

the whole cycle of the reaction f6p + g3p ↔ e4p + xu5p, which exchanges two first carbons of f6p and xu5p. This exchange is also coupled with exchange of four last carbons of f6p and e4p, and three last carbons of xu5p and g3p.

Three reactions shown in Figure 7a, 7b and 7c, catalysed by the same enzyme, create 6 different isotope exchange fluxes corresponding to forward and reverse directions of these reactions. These three reactions are formed by three half reactions, which could independently contribute to isotope exchange, as Figure 8 shows:

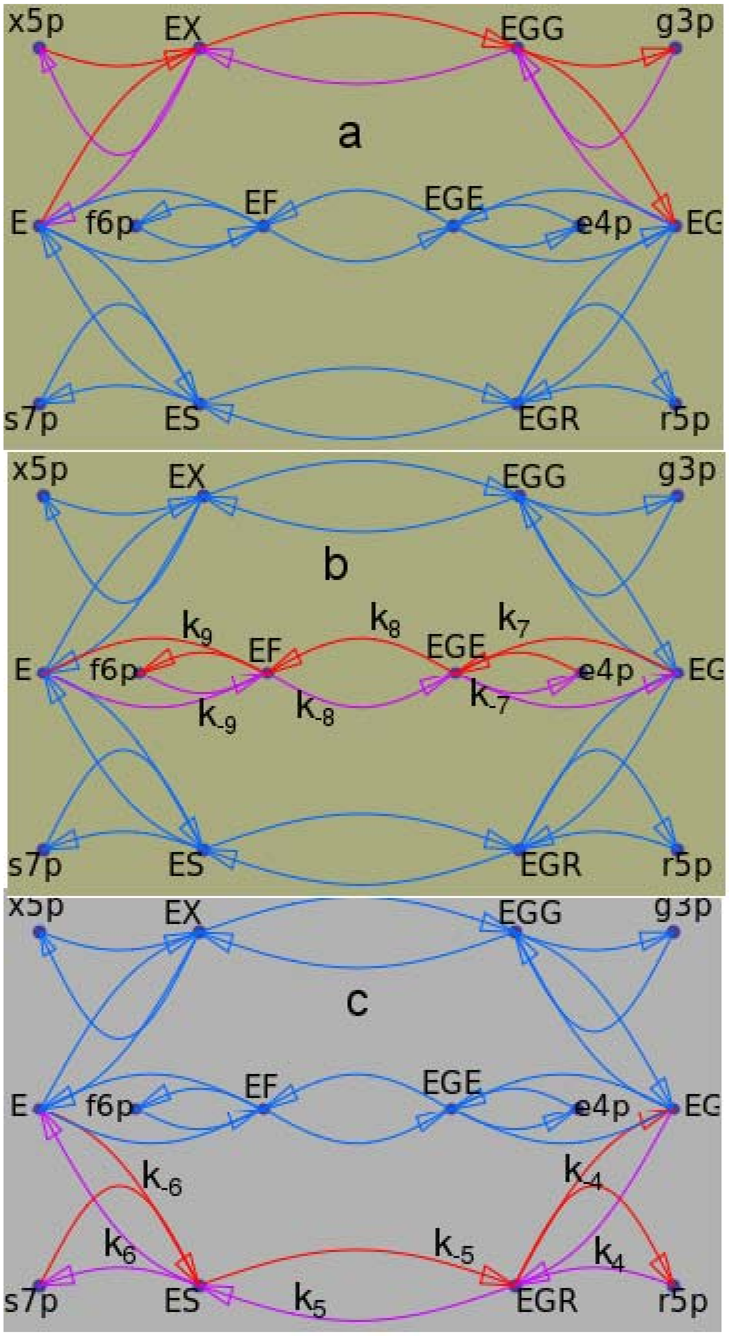

Figure 8. Isotope exchange catalyzed by transketolase reaction without a change of substrate concentration. This exchange proceeds when, after performing only the half of reaction, enzyme returns back to initial free form (E) through the same states in reverse direction (red and magenta paths). **(a)**, exchange between xu5p and g3p; **(b)**, exchange between f6p and e4p; **(c)** exchange between s7p and r5p.

Thus, transketolase catalyzes 9 different isotope exchange fluxes (forward and reverse through the whole cycle for three reactions and three half-reactions performing isotope exchange without total concentration change). The expressions for all these fluxes, which were necessary for the simulation of isotope exchange described below, were obtained by solving the equations describing the transitions of atoms of specific origin, similar to (9) and (10) for aldolase.

The expressions for reaction rates through elementary rate constants and substrate concentrations were obtained using King-Altman graphical rules. The final expressions for all the fluxes for aldolase, transaldolase and transketolase are present in the C++ source file tk.cpp, in Annex 1.

Module for simulation of isotopomer distribution

If cells are incubated with stable isotope labeled substrates, the same reactions, which are schematically shown in Figure 1, distribute also the isotopomers of metabolites. Thus, system (1) is valid in this case, but the variables (concentrations of metabolites) could be considered in this case as a sum of isotopomer concentrations and the reaction rates could be considered as a sum of transformations of all isotopomers in a given reaction. For a substrate consisting of n carbon atoms the total metabolite concentration is presented in our module for the simulation of isotopomer distribution as a sum of 2^n ^{13}C isotopomers.

Classical kinetic models do not account for the changes in carbon skeleton of molecules in described biochemical reactions. To account for isotopomer distribution, the specific ways of splitting and reformation of carbon skeleton are critical; the positions of carbon atoms, which could be labeled or not labeled, must be reflected in the model and the specific transformations of carbon skeleton in described reactions must be simulated. Isotopomers are presented as sets of “0” (for not labeled ^{12}C) and “1” (for labeled ^{13}C), which in the same time represent integers in binary form. For instance, for a metabolite A containing three carbon atoms all possible isotopomers are:

000, 001, 010, 011, 100, 101, 110, 111 (21)

If a reaction transforms carbon skeleton of A, the program performs respective changes with the model representatives of isotopomers. If, for instance, a reaction transforms A in B binding one not labeled carbon atom to the first carbon of A, it will create a set of fluxes converting (20) into the isotopomers of B:

0000, 0001, 0010, 0011, 0100, 0101, 0110, 0111 (21a)

The values of these fluxes are

$$v_{ia} = A_{ia}*V_t/A_t \quad (22)$$

here the integer number ia is the position of an isotopomer in the array (21), which coincides with its binary representation (isotopomer 000 occupies position 0; ...; 111, (7 in decimal), occupies position 7). v_{ia} is an efflux of isotopomer ia being transformed, and in the same time it is an influx to the isotopomer, which is the result of transformation (in the present example it is four-carbon isotopomer of B, 0ia). A_{ia} is the concentration of the isotopomer being transformed; V_t is the total reaction rate and A_t is total metabolite concentration, taken from the result of respective kinetic model execution. Sum of all v_{ia} gives V_t and sum of all A_{ia} gives A_t. This is the connection of kinetic model with the module for isotopomer distribution analysis. V_t and A_t are calculated by kinetic model based on the accepted mechanisms of enzyme reactions and regulation, and then for isotopomer distribution analysis the particular isotopomer fluxes v_{ia} are computed to be proportional to the concentration A_{ia} of respective isotopomer.

Initially all intermediates are assumed to be not labeled and the concentrations of isotopomers in initially added substrate is defined by experimental procedure.

If the reaction A → B is the only one, where A participates, the system of differential equation describes the evolution of isotopomer-substrate and isotopomer-product:

$$A_{ia}' = -v_{ia} \quad (23)$$

$B_{ib}' = v_{ia}$

where vi is defined by (22). Based on the rule of isotopomer transformation in a specific reaction A → B, or (21) → (21a), which resides in adding a zero before each A isotopomer number, the program automatically calculates the derivatives for all the isotopomers of A and B. To solve the system of thus constructed differential equations we implemented various standard methods (Press et al, 2002)

In the present above example the positions of isotopomer-substrate and isotopomer-product are the same, but if they are different, the algorithm accounts the difference. If, for instance, in the same transformation of three-carbon A in four-carbon B a labeled carbon atom is bound, (21) would be transformed respectively in

1000, 1001, 1010, 1011, 1100, 1101, 1110, 1111 (21b)

i.e. ib = 1000 + ia (in binary notation)

In real metabolic system the same metabolite (and isotopomers) participates in several reactions as a substrate or product. In this case the expressions for derivatives (23) contain more terms corresponding to the number of reactions in which they participate. The program passes through all the reaction in the simulated metabolic pathway and adds terms to the expressions for corresponding isotopomers.

In the pathway, which we simulated (Figure 1) there are several reactions types with the specific rules of transformation of isotopomer-substrate into isotopomer-product. We developed specific algorithms for automatic determination of isotopomer-product for each isotopomer-substrate for each reaction type. These algorithms constitute a library of functions, which could be used for by the automated procedure of construction of ODE system describing the dynamics of isotopomer transformations.

These algorithms are described next.

Reactions without carbon skeleton change. Function "input"

This function is used for automated construction of reaction rate terms describing the transitions from glucose to glucose 6-phosphate (hexokinase), from glucose 6-phosphate to glycogen, from fructose 6-phosphate to fructose bisphosphate (PFK), from glyceraldehyde 3-phosphate to phosphoenol pyruvate, from phosphoenol pyruvate to pyruvate (PK), from citrate to glutamate. It is an attribute of class, which includes an array of concentrations for all isotopomers of metabolite (A) and the functions, which simulate reactions, where this metabolite is used as a substrate.

It takes input parameters: a class of metabolite-product (B), the ratio of total forward outflux to total substrate concentration (V_{tf}/A_t, see (22)) obtained from respective classical kinetic model output, and the ratio of total reverse influx from metabolite-product to total product concentration (V_{tr}/B_t).

For each isotopomer-substrate it computes

$$v_i = A_i*V_{tf}/A_t - B_i*V_{tr}/B_t \quad (24)$$

subtracts v_i from the derivatives of isotopomers-substrate A_i and adds it to the derivatives of isotopomers-product B_i. In this particular case isotopomer-substrate and isotopomer-product are the same, because no change in carbon skeleton takes place.

Decarboxylation of first carbon. Function “g6pdh”

This function is used for automated construction of reaction rate terms for a reaction resulted in splitting the bond between first and second carbon atoms and the release of first carbon atom. In the simulated pathway it was used to describe the transition from g6p to the pool of pentose phosphates and also from pyruvate to acetil coenzyme A (PDH).

It takes input parameters: a class containing array of substrate isotopomer concentrations, a class containing array of product isotopomer concentrations, and the ratio of total forward outflux to total substrate concentration (V_{tf}/A_t, see (22)) obtained from respective classical kinetic model output.

For each isotopomer-substrate it computes

$$v_i = A_{ia}*V_{tf}/A_t \qquad (25)$$

subtracts v_i from the derivatives of isotopomers-substrate A_i and adds it to the derivatives of isotopomers-product B_{ib}, where ib is obtained by removing the left digit from the number of ia in binary notation. If A contains n carbon atoms, B contains (n-1) atoms and using C++ the removal of first digit can be expressed as[2]:

$$ib = (ia\&((1<<(n-1))-1)) \qquad (26)$$

Decarboxylation of last carbon. Function “decarb”

This function simulates reactions resulting in the release of last carbon atom of substrate molecule. In our model it was applied for the simulation of decarboxylation of oxaloacetate into phosphoenolpyruvate (PEPCK) and of a block of reactions in TCA cycle which include decarboxylation of a-ketoglutarate and production of malate.

Being a member of class-substrate (A) it takes as parameters class-product (B) and the ratio of total reaction rate and substrate concentration (V_t/A_t), obtained from classical kinetic model. It computes reaction rate (v_i) for each isotopomer of A using equation (26), subtracts it from the derivatives of isotopomers A_i and adds it to the derivatives of isotopomers B_{ib}, where ib is obtained by removing the right digit from the number of ia in binary notation. This rule in C++ can be expressed as:

$$ib = (ia>>1) \qquad (27)$$

Pyruvate carboxilation. Function “carb”

This function simulates reactions performing changes in the carbon skeleton oppsite to those simulated by "decarb": binding one carbon to the last position in substrate molecule. It was used to simulate pyruvate carboxylase reaction.

As "decard", it also is a member of class-substrate (A) it takes as parameters class-product (B) and the ratio of total reaction rate and substrate concentration (V_t/A_t), obtained from classical kinetic model. It computes reaction rate (v_i) for each isotopomer of A using equation (26), subtracts it from the derivatives

---

2 In C++ (and C) operator '<<' signifies bitwise shift left of the left operand by the number of positions specified by right operand, 1<<2 gives 100 in binary or 4 in decimal. '>>' is bitwise shift right, & is bitwise 'AND', '|' is bitwise 'OR'.

of isotopomers A_i and adds it to the derivatives of isotopomers B_{ib}, where ib is obtained by addition "0" to the right of number of ia in binary notation. In C++ this rule can be expressed as:

ib = (ia<<1) (28)

TCA cycle. Function “csyn”

This function simulates a block of reactions starting from citrate synthase, decarboxylation of isocitrate and production of a-ketoglutarate. For each of four isotopomers of acetil part (i) it calculates the reaction rate for interaction with eact of 16 isotopomers of oxaloacetate (j):

$$v_{ij} = [ac]_i*[oaa]_j V_t/([accoa]_t*[oaa]_t) \quad (29)$$

Here V_t, $[accoa]_t$ and $[oaa]_t$ are total reaction rate, accoa and oaa concentrations taken from classical kinetic model. The program subtracts this rate calculating derivatives of isotopomers $[ac]_i$ and $[oaa]_j$ and adds this rate to the derivative of isotopomer-product, which digital representation (ic) is determined as follows.

Since two atoms of acetil became first two atoms of a-ketoglutarate, the program takes i in binary form as a beginning of isotopomer product. Since the first carbon atom originated from oxaloacetate is removed in the reaction of decarboxilation of isocitrate, the program removes respective digit in the binary representation of oaa isotopomer (j) and put the rest (3 digits) after the number i of acetil isotopomer. This procedure was described in C++ as

ic = ((i<<3)&(j&7)) (30)

Thus, for this bimolecular reaction the progran calculates 4*16 reaction rates for each couple of interacting isotopomers, determines the digital representation of isotopomer-product, subtrats the calculated rate from the derivatives of substrates and adds it to the derivative of product.

Symmetry of fumarate. Function “symm”.

Fumarate is symmetrical molecule. This means that there is no means to distinguish carbons 1 and 4 or 2 and 3. If a fumarate molecule labeled in position 1 is transformed in malate, this latter has 50% probability to be labeled in position 4. Vice versa, fumarate labeled in position 4 has 50% probability to be transformed in malate labeled in position 1. To account for such result of symmetry of fumarate, we apply to the isotopomers of fumarate a function, which equals the concentrations of isotopomer pairs of fumarate, which are symmetrical with respect to the other one in the pair. Specifically, the program makes equal the concentrations of the following isotopomers, keeping their sum intact: 0001 and 1000, 0010 and 0100, 0011 and 1100, 0101 and 1010, 0111 and 1110, 1011 and 1101.

Fluxes in aldolase reaction. Functions “split” and "splnvs".

Function “split” calculates isotope exchange induced by forward (v_f) and reverse (v_r) fluxes when aldolase passes through the whole catalytic cycle (equations (12) and (15)). For each isotopomer i of fructose

bisphosphate it calculates the forward flux of its splitting to trioses and reverse flux of its formation from trioses:

$$v_i = [fbp]_i * v_f/[fbp]_t - [t3p]_{i1} * [t3p]_{i2} * v_r/[t3p]_t^2 \qquad (31)$$

Here and below index t indicates the total concentrations (here fbp and t3p) obtained from classical kinetic model solution. The program subtract v_i from the derivative of $[fbp]_i$ and adds it to the derivatives of $[t3p]_{i1}$ and $[t3p]_{i2}$.

The isotopomers i1 and i2 are defined as follows. Since i, reference number of fbp isotopomer, in the same time is the digital representation of isotopomer, the numbers i1 and i2 are obtained by splitting of i, which simulates the splitting of real isotopomer by aldolase.

When a molecule of fbp is split into two trioses, the last three carbons does not change their order, 4th becomes 1st in triose, 5th becomes 2nd, 6th becomes 3^{d}.

This rule could be implemented in C++ as

`i2= (i&7)` (32)

First part of the fbp molecule changes the order when it is transformed into triose: 1st atom of fbp becomes 3d in triose and 3d in fbp becomes 1st in triose. This rule could be implemented in C++ as

first digit of fbp is `((i>>5)&1);`

second digit of fbp is `((i>>4)&1);`

third digit of fbp is `((i>>3)&1);`

They are reordered in the produced triose as

`i1 = (((i>>3)&1)<<2)|(((i>>4)&1)<<1)|((i>>5)&1).` (33)

Thus, for each isotopomer i of fbp, the program defines isotopomer-products using the rules (32) and (33) and calculates the contributions of aldolase reaction to their derivatives according to (31).

}

Function "spInvs" calculates pure isotope exchange without a change of total concentrations induced by a partial flux (v_e) defined in (20). For each isotopomer i of fructose bisphosphate it calculates the forward flux of its splitting to trioses and reverse flux of its formation from trioses:

$$v_i = [fbp]_i * v_f/[fbp]_t \qquad (34)$$

The program subtract v_i from the derivative of $[fbp]_i$ and adds it to the derivatives of produced triose $[t3p]_{i1}$. Since the first part of molecule is released and the positions of carbon atoms in released triose are reverse compared to those in fbp, the binary representation of produced triose is recalculated in the same way as in (33).

The flux (v_e) does not change the total concentration of fbp and trioses. Thus, the same flux must bring back the same amount of fbp and taking the same amount of trioses as (34). However this flux back combines various triose isotopomers with the same last part of the isotopomer i of fbp. Therefore the reverse flux is divided to 8 subfluxes from each triose isotopomers:

$v_{ij} = [t3p]_j * v_i / [t3p]_t$ (35)

this flux is subtracted from the derivative of $[t3p]_j$ and added to the derivative ij of fbp, which is defined as follows. Last part of the molecule of new formed isotopomer ij is the same as that of isotopomer i. First part of it is the molecule of isotopomer j of triose, in which the positions of carbon atoms are renumbered in reverse order. This rule was implemented in C++ as:

`ij = (((j>>2)&1)<<3)|(((j>>1)&1)<<4)|(j&1)|(i&7).` (36)

Fluxes in transaldolase and transketolase reactions. Functions “tk” and "invis".

Like in the simulation of aldolase reaction, there are two different functions, which simulate transaldolase and transketolase reactions. Function "tk" simulates isotope exchange related with a flux through the whole reaction cycle, which could change also the total concentrations of metabolites. "tk" takes as parameters the classes of two substrates (ket1, ald1) and two products (ald2, ket2), the value of one of the total fluxes through the whole reaction cycle (v) described in the paragraphs 3.2 or 3.3, and integer flag, which indicates whether it is transaldolase (0), or transketolase (1) reaction.

This function calculates the rate of reaction between each isotopomer of substrate-ketose (i1) with each isotopomer of substrate-aldose (j1):

$v_i = [ket1]_i * [ald1]_j * v / ([ket1]_t * [ald1]_t)$ (37)

This reaction rate is subtracted from the derivatives of isotopomers $[ket1]_i$ and $[ald1]_j$, and added to the derivatives of products of these isotopomers interaction $[ket2]_{i2}$ and $[ald2]_{j2}$. The isotopomers i2 and j2 are defined by simulation of the splitting of substrates and reformation of products in accordance with reaction mechanism.

If ket1 contains n carbon atoms and the function simulates transaldolase reaction (split between third and fourth carbon atoms and release the last part of the molecule as new formed aldose), then for new aldose is defined using C++:

`j2 = (i&(1<<(n-3)-1))` (38)

If aldose-substrate contains m carbons, transaldolase binds them to the three-carbon fragment persisting in the active site, thus forming (m+3)-carbon ketose product. Thus produced isotopomer of ket2 is defined using C++:

`i2=(((i>>(n-3))<<m)|j)` (39)

If function "tk" simulates transketolase reaction (this is defined by parameter flag, in this case it is assigned to 1), which splits ketose-substrate between second and third carbon atoms, the expression for reaction rate (37) remains the same, but the isotopomers-products are described slightly differently, in accordance with a different reaction mechanism.

`j2 = (i&(1<<(n-2)-1))` (38a)

`i2=(((i>>(n-2))<<m)|j)` (39a)

Function "invis" simulates a partial forward and reverse flux, which does not change the total metabolite

concentrations, but only exchanges a part of ketose molecule with aldose that could be produced by the ketose splitting. "invis" takes as parameters the classes of two substrates (ket, ald), the value of a total flux of exchange between ketose and produced aldose (v) described in the paragraphs 3.2 or 3.3, and integer flag, which indicates whether it is transaldolase (0), or transketolase (1) reaction.

This function calculates the rate of reaction between each isotopomer of substrate-ketose (i1) with each isotopomer of substrate-aldose (j1):

$$v_i = [ket1]_i*v/ [ket1]_t \quad (40)$$

This reaction rate is subtracted from the derivatives of isotopomers $[ket1]_i$ and added to isotopomer of produced aldose (j) defined in accordance with $[ket1]_i$ splitting by transaldolase:

j=(i & ((1<<(n-3)) - 1)) (41)

or transketolase:

j=(i & ((1<<(n-2)) - 1)) (42)

The flux does not change total concentration of ketose i aldose, because it brings back to the pools the same amount but with different isotopomer composition. This flux back combines various aldose isotopomers with the same first part of the isotopomer i of ketose. Therefore the reverse flux is divided to the subfluxes from each aldose isotopomer.:

$$v_{ij} = [ald]_j*v_i /[ald]_t \quad (43)$$

this flux is subtracted from the derivative of $[ald]_j$ and added to the derivative ij of ket, which is defined as follows. First part of the molecule of new formed isotopomer ij is the same as that of isotopomer i. Last part of it is the molecule of isotopomer j of aldose. This rule was implemented in C++ as:

ij = (((i>>(n-2))<<(n-2))|j) (in case of transketolase) (44)

ij = (((i>>(n-3))<<(n-3))|j) (in case of transaldolase) (45)

Dilution of mass isotopomers.

Often mass isotopomer distribution is measured in ribose derived from RNA. For the analysis of such data it is convenient to consider it as consisted of two parts: one part is totally unlabeled initial ribose from the nucleotides persisted in cells before incubation, and the other is synthesized *de novo* during incubation with labeled glucose, in which the distribution of isotopomers considers with that in free ribose 5-phosphate synthesized in pentose phosphate pathway. Using the above described algorithms the program calculates the isotopomer distribution in free ribose 5-phosphate, which is different from the distribution in RNA ribose because of dilution of latter by the fraction initially unlabeled. To compare the results of calculation with the experimental data the calculation must account for the dilution. If the program calculates realistic distribution of mass isotopomers in free ribose 5-phosphate, the following relationship takes place:

$$m0_e=(C0_c+C0_i)/(C_t+C0_i); \quad (46)$$

where $m0_e$ is a measured fraction of unlabeled ribose, $C0_c$ is calculated concentration of free unlabeled ribose 5-phosphate, $C0_i$ is an imaginary concentration on unlabeled ribose, which dilutes the calculated

ribose 5-phosphate to the same extent as *de novo* synthesized RNA ribose is diluted by initially present fraction. C_t is calculated total concentration of free ribose 5-phosphate. Division of nominator and denominator in the right hand side of (46) by C_t gives

$$m0_e=(m0_c+d)/(1+d); \qquad (47)$$

where $m0_c=C0_c/C_t$ is the calculated fraction of unlabeled free ribose 5-phosphate, and $d=C0_i/C_t$ is the fraction of initially present unlabeled ribose in RNA with respect to the fraction synthesized *de novo*. From (47) d could be expressed as

$$d=(m0_e-m0_c)/(1-m0_e) \qquad (48)$$

Once the dilution is defined based on the calculated and measured unlabeled fractions m0, its contribution to the values of the other mass isotopomer fractions (m1, m2, etc) can be defined.

$$m1_d=C1_c/(C_t+C0_i);\ m2_d=C2_c/(C_t+C0_i);\ \text{etc.,} \qquad (49)$$

where subindex d indicates that dilution was taken into account. Dividing the nominator and denominator by C_t gives

$$m1_d=m1_c/(1+d);\ m2_d=m2_c/(1+d);\ \text{etc.,} \qquad (50)$$

The calculation of dilution (48) depends on the calculated value of unlabeled fraction of free ribose 5-phosphate. However it is an objective of simulation, to find a set of parameters, which minimizes the difference between calculated and measured values. It is not known in advance, whether the calculation of mass isotopomer distribution of free ribose 5-phosphate is correct or not. The solution of this problem is in the correspondence between measured and calculated values for the other mass isotopomers. The dilution is computed using only the fraction m0, and the other fractions are obtained without using experimental information. We consider, that the isotopomer distribution and dilution are defined correctly if the other fractions, calculated based on (50), are close to the measured values.

The structure of program

The whole program is constructed in the way that the classical kinetic model, expressed by equations (1), represents a separate module, which, in principle, can be compiled and executed independently from the parts, simulating isotopomer distribution. After the termination of this execution, the program calls the module, which calculates additional fluxes described in section 3. These fluxes together with concentrations and fluxes computed directly by classical kinetic model provide interface with the module that computes derivatives of all isotopomers by calling the functions described in section 4. The obtained system of equations for the derivatives of isotopomers can be solved by a usual numerical ODE solving procedure. We implemented several methods provided for C++ by Press et al (2002). The solvers calls the module for recalculation of numerical values of isotopomer derivatives as it would call any usual system of ODE.

The system of ODE for isotopomers in fact reflects the system (1), which describes the evolution of total concentrations. The difference is that concentrations represent vectors of isotopomers and the reaction rates (v) are calculated automatically for each isotopomer by calling specific functions described in section 4.

If the model simulates the dynamics of isotopomer distribution when, in general, metabolism is in steady state, the steady state fluxes and concentrations could be computed by a single ran of kinetic model, and then isotopomer dynamics can be calculated using the defined total concentration and fluxes.

If the model simulates dynamics of isotopomer distribution during perturbations of metabolic state, the computation is organized in such a way that kinetic simulations of short time intervals are followed by the same simulations of isotopomer dynamics. The maximal value of such time interval is defined by the rate of change of metabolic fluxes and it could be determined by robustness of solution for isotopomer dynamics.

Since kinetic model is used by the program as a separate module, various kinetic models could be attached to the part that simulates isotopomer distributions. In other words, the isotopomer distribution library can be used for connection with various kinetic models to analyse respective specific problems where ^{13}C tracer was used.

Below we describe two examples of application of our software for the analysis of isotopomer distributions in different objects with different kinetic models, and also an example of adopting an arbitrary model, found in SBML models database, to the analysis of isotopomer distributions.

References

1. Wiechert W, Wurzel M. Metabolic isotopomer labeling systems. Part I: global dynamic behavior. Math Biosci. 2001, 169, 173-205.

2. Isermann N, Wiechert W. Metabolic isotopomer labeling systems. Part II: structural flux identifiability analysis. Math Biosci. 2003,183, 175-214.

3. Chandel NS, et al (2000). Role of oxidants in NF-kappa B activation and TNF-alpha gene transcription induced by hypoxia and endotoxin. J Immunol 165:1013-1021.

4. Li C, Jackson RM (2002) Reactive species mechanisms of cellular hypoxia–reoxygenation injury. *Am J Physiol: Cell Physiol* 282: C227–C241

5. Pisarenko O, Studneva I, Khlopkov V, Solomatina E, Ruuge E (1988) An assessment of anaerobic metabolism during ischemia and reperfusion in isolated guinea pig heart. *Biochim Biophys Acta* 934: 55-63.

6. Rabinovich RA, et al (2001). Reduced muscle redox capacity after endurance training in COPD patients. Am J Respir Crit Care Med 164:1114-1118

7. Rabinovich RA, et al (2007). Mitochondrial dysfunction in COPD patients with low body mass index. Eur. Respir. J. 29, 643-650.

8. Selivanov VA, et al (2004). An Optimized Algorithm for Flux Estimation from Isotopomer Distribution in Glucose Metabolites. Bioinformatics 20, 3387-3397.

9. Selivanov VA, et al (2005). Rapid simulation and analysis of isotopomer distributions using constraints based on enzyme mechanisms: an example from HT29 cancer cells. Bioinformatics 21, 3558–3564.

10. Selivanov VA, et al (2006). Software for dynamic analysis of tracer-based metabolomic data: estimation of metabolic fluxes and their statistical analysis. Bioinformatics, 22, 2806-2812.

11. Press WH, Flannery BP, Teukolsky SA, Vetterling WT (2002) Numerical Recipes in C: The Art of Scientific Computing. Cambridge University Press, NY.

12. A. Cornish-Bowden (1979) *Fundamentals of Enzyme Kinetics,* Butterworths, London & Boston (220 pp.)

13. Mulquiney PJ, Kuchel PW. Model of 2,3-bisphosphoglycerate metabolism in the human erythrocyte based on detailed enzyme kinetic equations: equations and parameter refinement. Biochem J. 1999 Sep 15;342 Pt 3:581-96.

14. McIntyre LM, Thorburn DR, Bubb WA, Kuchel PW. Comparison of computer simulations of the F-type and L-type non-oxidative hexose monophosphate shunts with 31P-NMR experimental data from human erythrocytes. Eur J Biochem. 1989 Mar 15;180(2):399-420.

15. Hucka M, Finney A, Sauro HM, Bolouri H, Doyle JC, Kitano H, Arkin AP, Bornstein BJ, Bray D,

Cornish-Bowden A, Cuellar AA, Dronov S, Gilles ED, Ginkel M, Gor V, Goryanin II, Hedley WJ, Hodgman TC, Hofmeyr JH, Hunter PJ, Juty NS, Kasberger JL, Kremling A, Kummer U, Le Novère N, Loew LM, Lucio D, Mendes P, Minch E, Mjolsness ED, Nakayama Y, Nelson MR, Nielsen PF, Sakurada T, Schaff JC, Shapiro BE, Shimizu TS, Spence HD, Stelling J, Takahashi K, Tomita M, Wagner J, Wang J; SBML Forum. The systems biology markup language (SBML): a medium for representation and exchange of biochemical network models. Bioinformatics. 2003 Mar 1;19(4):524-31.
